# Factors predicting access to medications for opioid use disorder for housed and unhoused patients: A machine learning approach

**DOI:** 10.1371/journal.pone.0308791

**Published:** 2024-09-27

**Authors:** Aaron Esguerra, Thomas J. Weinandy

**Affiliations:** 1 College of Human Medicine, Michigan State University, Grand Rapids, Michigan, United States of America; 2 Upside, Washington, District of Columbia, United States of America; University of New Mexico Health Sciences Center, UNITED STATES OF AMERICA

## Abstract

**Background:**

Opioid use disorder (OUD) is a growing public health crisis, with opioids involved in an overwhelming majority of drug overdose deaths in the United States in recent years. While medications for opioid use disorder (MOUD) effectively reduce overdose mortality, only a minority of patients are able to access MOUD; additionally, those with unstable housing receive MOUD at even lower rates.

**Objective:**

Because MOUD access is a multifactorial issue, we leverage machine learning techniques to assess and rank the variables most important in predicting whether any individual receives MOUD. We also seek to explain why persons experiencing homelessness have lower MOUD access and identify potential targets for action.

**Methods:**

We utilize a gradient boosted decision tree algorithm (specifically, XGBoost) to train our model on SAMHSA’s Treatment Episode Data Set-Admissions, using anonymized demographic and clinical information for over half a million opioid admissions to treatment facilities across the United States. We use Shapley values to quantify and interpret the predictive power and influencing direction of individual features (i.e., variables).

**Results:**

Our model is effective in predicting access to MOUD with an accuracy of 85.97% and area under the ROC curve of 0.9411. Notably, roughly half of the model’s predictive power emerges from facility type (23.34%) and geographic location (18.71%); other influential factors include referral source (6.74%), history of prior treatment (4.41%), and frequency of opioid use (3.44%). We also find that unhoused patients go to facilities that overall have lower MOUD treatment rates; furthermore, relative to housed (i.e., independent living) patients at these facilities, unhoused patients receive MOUD at even lower rates. However, we hypothesize that if unhoused patients instead went to the facilities that housed patients enter at an equal percent (but still received MOUD at the lower unhoused rates), 89.50% of the disparity in MOUD access would be eliminated.

**Conclusion:**

This study demonstrates the utility of a model that predicts MOUD access and both ranks the influencing variables and compares their individual positive or negative contribution to access. Furthermore, we examine the lack of MOUD treatment among persons with unstable housing and consider approaches for improving access.

## Introduction

Opioid use disorder (OUD) is a growing public health emergency, with opioids involved in 81.8% of all drug overdose deaths in the United States (42,065 in 2022 alone) [1]. Treatment via medications for opioid use disorder (MOUD) (e.g., methadone, buprenorphine, naltrexone) is shown to be a safe and effective therapeutic approach and demonstrably lowers the risk of death from overdose [2, 3].

Despite this, a minority of individuals with OUD report receiving these medications [4]. Multiple factors influence access to MOUD. Homelessness is a notable barrier, associated with limited resources and support, a higher prevalence of physical and psychiatric comorbidities, and an unstable surrounding milieu [5–7]; furthermore, homelessness has been linked to increased rates of opioid overdose [8]. In the United States, the number of persons experiencing homelessness is on the rise [9], necessitating a close examination of housing insecurity in the context of OUD treatment.

With various biopsychosocial forces exerting complex multidirectional and overlapping effects, discerning their individual influence on MOUD access can inform effective policy. Machine learning analyses provide an advantage over classical statistical approaches by allowing for examination of such nuance, even in the setting of numerous input features and nonlinear interactions, and permit development of predictive models generalizable beyond the initial dataset [10]. Indeed, machine learning methods have been employed to predict development of OUD [11], opioid overdose [12], treatment adherence [13, 14], and post-treatment relapse [15]; with regards to predicting MOUD access, previous research utilized a tree-based machine learning analysis, although it centered on an intersectionality framework around racial disparities [16].

Persons experiencing homelessness are overrepresented in the population of individuals with OUD [17], yet research on this subset of the unhoused population is sparse. The current literature on MOUD access for the unhoused, a challenging population to study, has had to rely on qualitative analyses [5], smaller sample sizes [18], or employs traditional analytical methods [19]. Our study seeks to add to the literature by leveraging a modern machine learning approach to answer two research questions. First, we quantitatively assess and rank which factors predict MOUD access for all patients admitted for OUD. Second, we measure the discrepancies in MOUD access by patient housing status and identify the variable most associated with that disparity.

To address these questions, we train a gradient boosted decision tree algorithm to predict whether a patient admitted with OUD will receive MOUD. Then we calculate Shapley values to interpret our model and understand which features (i.e., variables) are most informative and whether they are associated with treatment or non-treatment. Shapley values have been used in other medical research, such as [20], who use them to quantify the role of diet on mortality risk. Gradient boost and Shapley values are ideal when used on a large number of observations, such as the 2019 Treatment Episode Data Set for Admissions provided by the Substance Abuse and Mental Health Services Administration which contains information on over 1 million admissions to treatment centers in the United States for various substance use disorders.

## Methods

### Machine learning model

Our machine learning model needed to be efficient with categorical data and a large number of observations while allowing for interaction effects between multiple features. We chose the gradient boosted decision tree algorithm for meeting these criteria; previous medical research demonstrates its utility in predicting outcomes of interest [12, 14]. Additionally, gradient boosted decision trees demonstrate superior performance compared to many other binary prediction models (e.g., logistic regression). It is more efficient than models with higher predictive power (e.g., artificial neural networks) and still highly interpretive [21]. Gradient boosted decision tree is also easily implemented, allowing for full replication of these results. All code is written in Python and available for public download at https://zenodo.org/records/12702494 while the source data is online [22]. We use the Extreme Gradient Boost (XGBoost) library to train the machine learning model and the SHapley Additive exPlanations (SHAP) library for model interpretation. Both libraries are widely cited in the academic literature, and as of January 2024, an exact search for “XGBoost” found 137,000 results on Google Scholar and “SHAP” identified 193,000 results.

Gradient boosted decision tree is a subclass of machine learning algorithms that begins by growing a single decision tree from training data (e.g., patients aged 35 and older, not on Medicaid, and going to a facility in California are predicted to receive treatment) and evaluates that decision tree based on actual outcomes. The algorithm then tries to grow an improved decision tree based on the errors of the previous tree and repeats this process for a defined number of trees as it “learns” the nuanced patterns in the data that predict treatment. This method does not provide causal answers but is still a useful way to identify associations between the independent variables and MOUD.

Gradient boosted decision trees also include many hyperparameters which are the features that define the algorithm training conditions (e.g., the number of branches in a decision tree, the number of trees it grows, the rate at which a tree learns from the previous tree, etc.). These hyperparameters impact the final structure and performance of the model, but it is uncertain which combination of hyperparameters is best. We therefore employ a Bayesian search algorithm that iteratively tries to predict a better combination of hyperparameters based on the results of previously tried combinations. We then selected the combination of parameters based on the set that produced a model with the highest area under the receiver operating characteristic curve (AUC).

### Feature importance

Once we have our trained gradient boost model, we interpret the model by calculating Shapley values for each variable. Shapley values come from the game theory literature to measure the average marginal impact each member has across all possible member combinations. In this case, we use it to measure the average amount of information each variable contributes toward making an accurate prediction. This feature (i.e., variable) importance allows us to quantify the predictive power of one variable relative to others within an algorithm. It also shows the direction of an effect, which for our model means that positive values indicate a variable that predicts MOUD access and negative values predict no MOUD access. Although Shapley values are not meaningful on their own, we can use their direction and relative values to identify which features are more predictive than others [23]. This allows us to both rank features by importance and calculate the relative importance of each variable out of 100%.

In our study, a high Shapley value indicates that a variable provides a strong signal that the machine learning model uses to predict which admissions will receive MOUD treatment. Conversely, a variable with a Shapley value of zero only contributes noise toward the prediction and is not associated with the outcome of MOUD treatment. Shapley values are a fundamental way to interpret how a predictive model like ours operates. It is critical for any machine learning algorithm that impacts patient health to be interpretable.

One major limitation of Shapley values is that they only identify a variable’s importance relative to other features, such that each variable’s Shapley value will change when a new variable is added or a current variable is removed from the model training process. This is less of a concern for our paper since we have a larger dataset of fifty-six features; however, we still caution against citing a specific feature importance value as absolute.

### Data

We examine data from the publicly available Treatment Episode Data Set for Admissions (TEDS-A), provided by the Substance Abuse and Mental Health Services Administration. TEDS-A provides demographic and clinical information of admissions to publicly-funded state-licensed or certified substance use treatment facilities as reported to state agency data systems annually across the United States. To avoid introducing potential confounders brought upon by the COVID-19 pandemic, we utilize the 2019 TEDS-A [22].

### Data processing

This paper is specifically interested in patients whose primary substance reported at admission (specified in TEDS-A as the variable SUB1) is an opioid. We therefore consider admissions in the dataset where SUB1 is either heroin, non-prescription methadone, or “other opiates and synthetics,” which TEDS-A defines as “buprenorphine, butorphanol, codeine, hydrocodone, hydromorphone, meperidine, morphine, opium, oxycodone, pentazocine, propoxyphene, tramadol, and other narcotic analgesics, opiates, or synthetics.”

We exclude several features from our analysis, including the year of admission (since they are all the same year) and case ID (as this is an index). The high number of unique values within each categorical variable (i.e., cardinality) can cause issues by decreasing the computational efficiency and increasing the likelihood of overfitting.

Additionally, the categorical variable DETNLF (detail not in labor force status) is a subset of the categorical variable EMPLOY (employed status). We subsume the child variable into the parent variable to create a new variable EMPLOY_DETNLF that details the various employed or unemployed status of an individual. Similarly, we combine the variable DETCRIM (detailed court referral) with the variable PSOURCE (primary referral source) to form the variable PSOURCE_DETCRIM.

Since we are specifically interested in whether individuals with OUD can access MOUD, we consider all admissions that report a primary substance of heroin, non-prescription methadone, or other opioids or synthetics. We also exclude all admissions whose access to MOUD is “Unknown” (7.3% of observations). This leaves a population of 524,134 admissions, from which we present descriptive statistics. Otherwise, we choose to keep all other features with a value of “Unknown.” Although there are many options for filling in missing data, we do not believe these values are missing at random. Assigning these entries to their own category of “Unknown” is an information preserving step that can then reveal patterns in the data. As shown later in the Results section, we find that many of these “Unknown” entries exhibit their own distinct patterns.

Before training our model, we randomly remove observations in the majority class until there are an equal number of admissions that do and do not receive MOUD. This ensures our model will not be overly biased toward the majority class, which in this case is not receiving MOUD. From there we build the gradient boosted decision tree model using 70% of the balanced dataset for training, 15% for testing, and 15% for validation.

We also encode all our features as either ordinal or pure categorical. For example, we define the variable AGE as having the order “Age12To14”, “Age15To17”, …, “Age55To64”, “Age65Plus”. This is useful for an algorithm like XGBoost that is making progressive splits to grow a decision tree and will split admissions by the ordered list (e.g., under 17 and over 18) instead of two randomly chosen sets of groups. Other features, such as an admission’s reported secondary substance use (SUB2), had no apparent order and were encoded as categorical.

### Descriptive statistics

After pre-processing, we identified 524,134 opioid admissions with a known MOUD treatment outcome (i.e., received MOUD or did not receive MOUD). This is the population we will consider for the remainder of the paper. 40.40% of these admissions with OUD received MOUD, showing a considerable shortfall in care with fewer than half of the admissions receiving the recommended care. We see a moderate difference in access according to the primary opioid used by the admitted patient. Admissions reporting heroin received MOUD in 40.20% of cases; admissions reporting non-prescription methadone received MOUD in 40.91% of cases; lastly, admissions reporting other opioids or synthetics received MOUD in 50.16% of cases.

**Fig 1:**
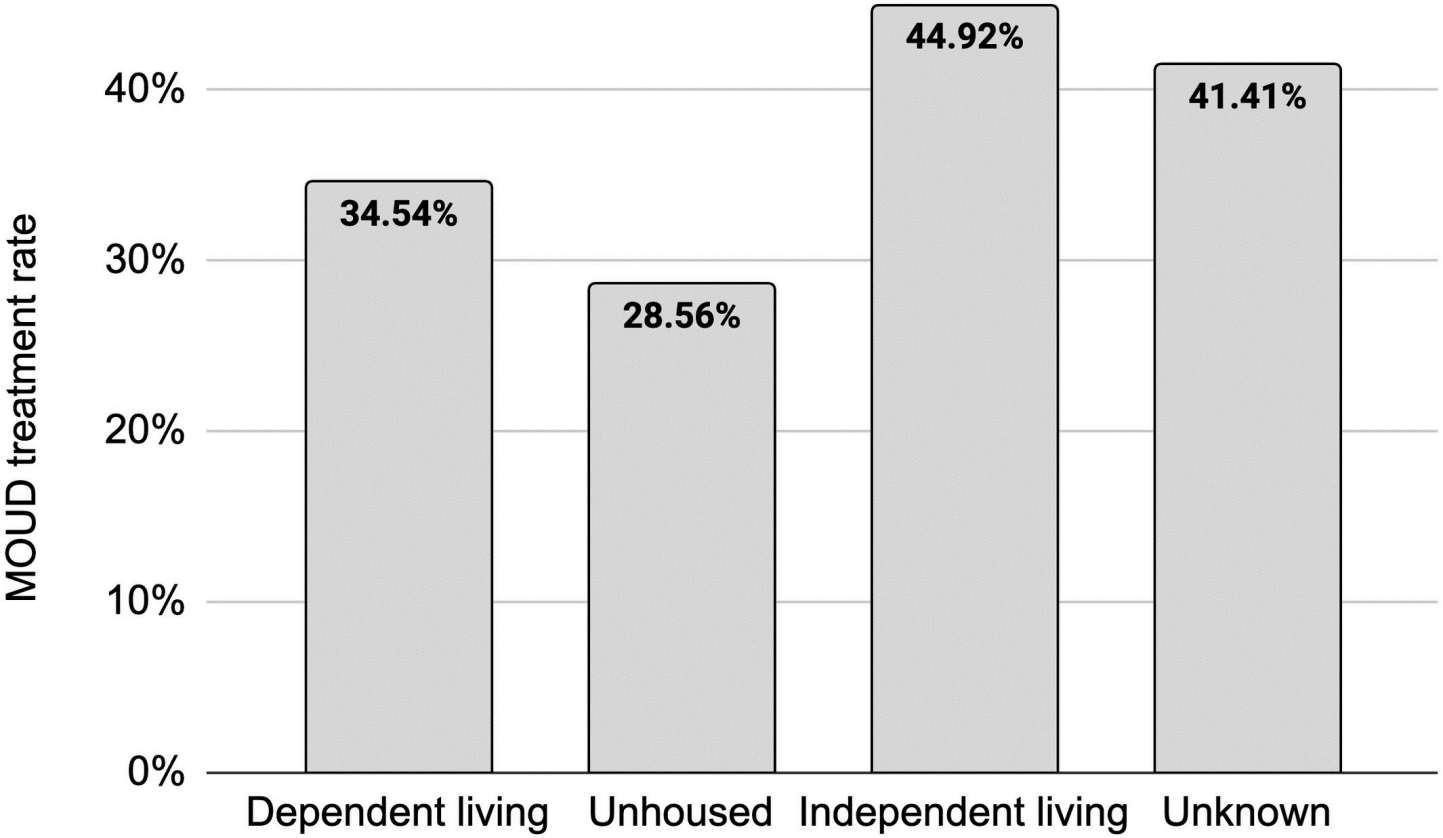
MOUD treatment rates by living status. Among all patients admitted to a service setting with OUD, those who live independently access MOUD at the highest rate (44.92%) while those who are experiencing homelessness access MOUD only 28.56% of the time.

We also turn to the role of housing status among opioid admissions and identify disparities in access to MOUD. As shown in Fig 1, we find wide differences in access according to the type of housing reported at admission. Admissions of patients who live independently (i.e., adults and adolescents living in a private residence and capable of self-care, including adult children living with parents) receive MOUD treatment in 44.92% of cases. Admissions of patients with dependent living arrangements (i.e., those living in a supervised setting such as a residential institution, halfway house, or group home as well as children living with parents, relatives, guardians, or foster care) receive MOUD treatment at a rate of 34.54% (23.11% lower than admissions of patients in independent living, a statistically significant difference at the 0.001 level). Admissions of patients experiencing homelessness (i.e., those with no fixed address, including those living in a temporary housing shelter) receive MOUD treatment at a rate of 28.56% (36.42% lower than admissions of patients in independent living, a statistically significant difference at the 0.001 level).

In total, there are 57 categorical features that describe our population of opioid admissions that further break down into 627 subgroups. S1 Appendix shows the MOUD treatment rates for each of these subgroups, including cross tabs by living arrangement. We also pick up on these disparities in access to care in the Results section.

## Results

### Machine learning model

The next step was to train a machine learning model to predict which opioid admissions would result in MOUD treatment. We used a gradient boost algorithm as implemented in the Python library XGBoost and trained the model on 70% of our dataset. We then validated hyperparameters on 15% of the data and tested the final model with a holdout test set from the remaining 15% of observations. See the S2 Appendix which describes the original and training datasets.

The best model was selected as that with the highest AUC (0.9411). The model was accurate for 85.97% of the test data when predicting all admissions with an estimated probability of receiving MOUD treatment equal to or greater than 50%. Since our algorithm was trained on a balanced dataset (i.e., an equal number of admissions treated and not treated with MOUD), an AUC of 0.5 would indicate a model is no better than random chance. Instead, our best model at an AUC of 0.9411 indicates it has reliably identified patterns in the data that predict which admissions for OUD are going to receive MOUD. The resulting model shows how observable characteristics of patients and the facilities they enter can be used to estimate the likelihood each admission has to receive MOUD.

### Feature importance

To predict MOUD access, the best gradient boost model had to learn which patterns in the data are associated with which outcome. We exploit this to identify the features (i.e., variables) our model finds most informative when making a prediction. We highlight the top twenty features in Table 1 and include the full list of 57 features in S3 Appendix.

**Table 1:** Top factors by feature importance.

Rank	Variable Name	Variable Short Definition	Variable Category	Share of Feature Importance
1	SERVICES	Type of treatment service/setting	Coordination of care	23.34%
2	STFIPS	Census state FIPS code	Geographic	9.45%
3	REGION	Census region	Geographic	7.02%
4	PSOURCE_DETCRIM	Referral source (combined with detailed criminal justice referral)	Coordination of care	6.74%
5	DIVISION	Census division	Geographic	5.77%
6	NOPRIOR	Previous substance use treatment episodes	Medical history	4.41%
7	CBSA2010	Core based statistical area (metro and micro areas)	Geographic	3.49%
8	FREQ1	Frequency of use (primary)	Substance use history	3.44%
9	DSMCRIT	DSM diagnosis (SuDS 4 SuDS 19)	Medical history	2.16%
10	HLTHINS	Health insurance	Economic	1.99%
11	AGE	Age at admission	Demographic	1.94%
12	FREQ_ATND_SELF_HELP	Attendance at substance use self-help groups in the past 30 days	Medical history	1.89%
13	PSYPROB	Co-occurring mental and substance use disorders	Medical history	1.81%
14	ARRESTS	Arrests in the past 30 days	Personal history	1.76%
15	PRIMPAY	Payment source, primary (expected or actual)	Economic	1.75%
16	DAYWAIT	Days waiting to enter substance use treatment	Coordination of care	1.68%
17	PRIMINC	Source of income/support	Economic	1.63%
18	SUB2	Substance use (secondary)	Substance use history	1.56%
19	EMPLOY_DETNLF	Employment status (combined with not in labor force)	Economic	1.53%
20	LIVARAG	Living arrangement	Economic	1.51%

Top features in the model that predict whether an admission has access to MOUD. Variable names are taken directly from the TEDS-A dataset and variable categories are assigned by the authors. The feature importance is calculated from Shapley values and normalized to add up to 100%. See the S3 Appendix for all 57 variables.

The ranked Shapley values (i.e., feature importance) in Table 1 reveal that the features with the highest predictive power are related to the treatment center and its location, including type of treatment service/setting (SERVICES) at 23.34%, census state FIPS code (STFIPS) at 9.45%, census region (REGION) at 7.02%, census division (DIVISION) at 5.77%, and Core Based Statistical Area (CBSA2010) at 3.49%. Consequently, this demonstrates that nearly half of the model’s predictive power comes from just five features that are determined before a patient even walks through the door.

**Fig 2:**
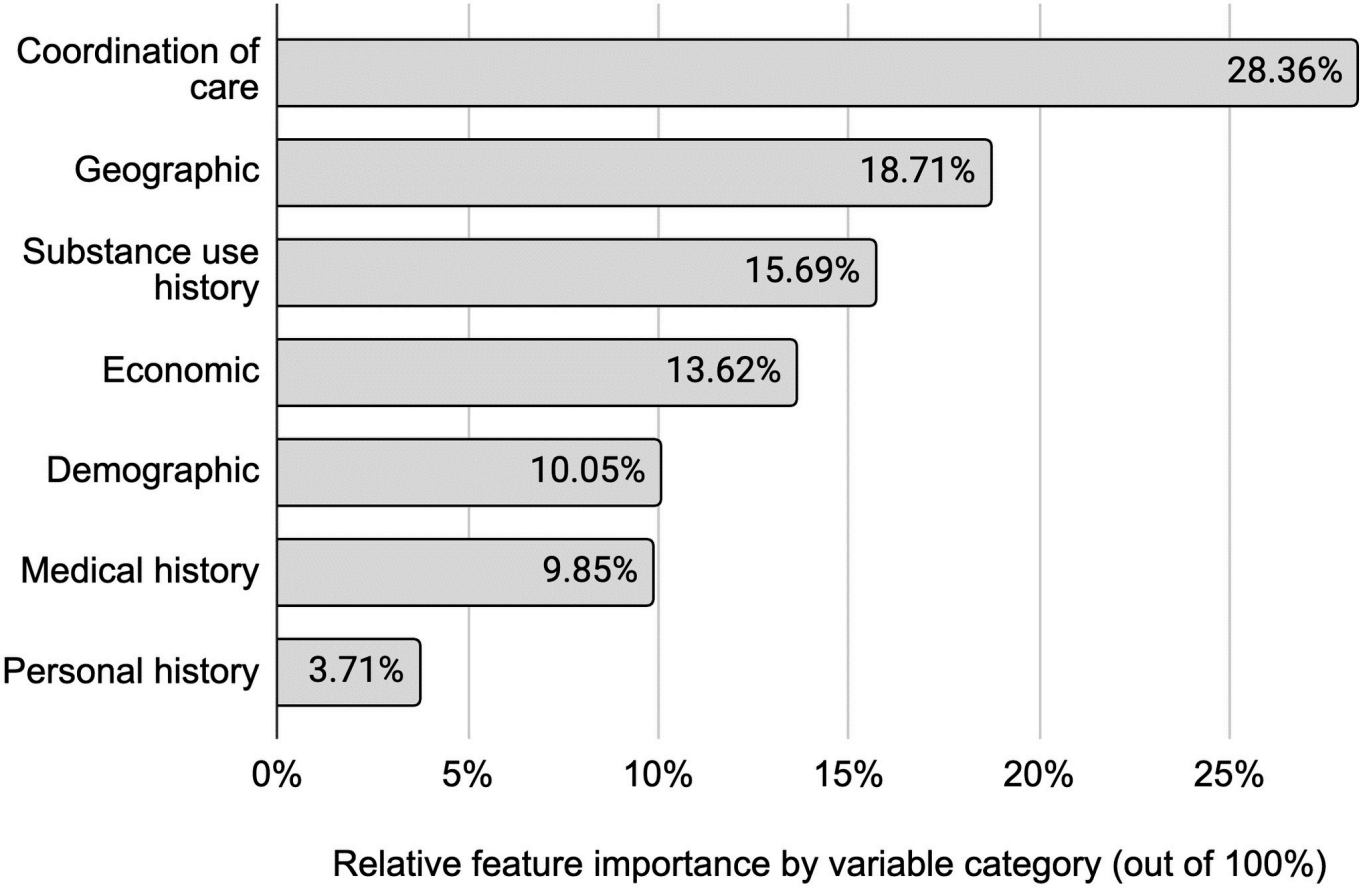
Aggregated feature importance by variable category. Nearly half of the features that predict MOUD access relate to coordination of care and geography of the service setting, indicating an opportunity for these facilities to improve treatment access for OUD patients. Variable categories were determined by the authors and aggregate the individual feature importance that describes how much each variable contributes to the model’s prediction. Feature importance is calculated from Shapley values and normalized to add up to 100%. See the S3 Appendix for a full variable list and their categories.

Since many of these features are related to each other, we also group them into feature categories to understand the themes of features that matter the most. These are shown individually in the Variable Category column in Table 1 and S3 Appendix. Additionally, aggregating the variable categories in Fig 2 reveals several insights about their combined feature importance. First, an admission’s coordination of care and geography are the two most informative categories of factors that predict MOUD treatment at 28.36% and 18.71%, respectively. Second, substance use history is the next most important variable category at 15.69% of the combined feature importance, indicating how an admission’s past relationship with substance use is associated with the type of treatment they receive. This is followed by economic conditions at 13.62% and a patient’s demographics at 10.05%. Finally, medical history (9.85%) and personal history (3.71%) have the lowest predictive impact on whether they receive OUD. However, other research shows that many of these factors are associated with accessing MOUD—e.g., race [24], age of first use [25], and gender [5]. We suspect some of these features impact treatment access indirectly, such as education (a component of the “personal history” feature category) predicting income (a component of the “economic” feature category), which then predicts MOUD access.

As a final exercise, we also dive deeper into how living arrangement predicts MOUD access. This is to 1) showcase how Shapley values can also be used to compare subgroups of a specific variable; and 2) highlight the disparities of treatment between housed and unhoused populations discussed in the literature [5, 6, 18].

As seen in Table 1, living arrangement as a variable (LIVARAG) is ranked 20th in terms of importance and only contributes 1.51% toward the model’s predictive capacity. This suggests that living status alone has only a modest effect on MOUD; however, living status is not determined in isolation, as Han and colleagues [19] find that unhoused patients with OUD are more likely to be male, of veteran status, unemployed, recently arrested, living in an urban center, and in the West. Building off this, the Shapley method we employ in our study splits the importance between all such variables, thus diluting the impact of living status. Next, we show how living status matters more for MOUD access than what is indicated by feature importance alone.

**Fig 3:**
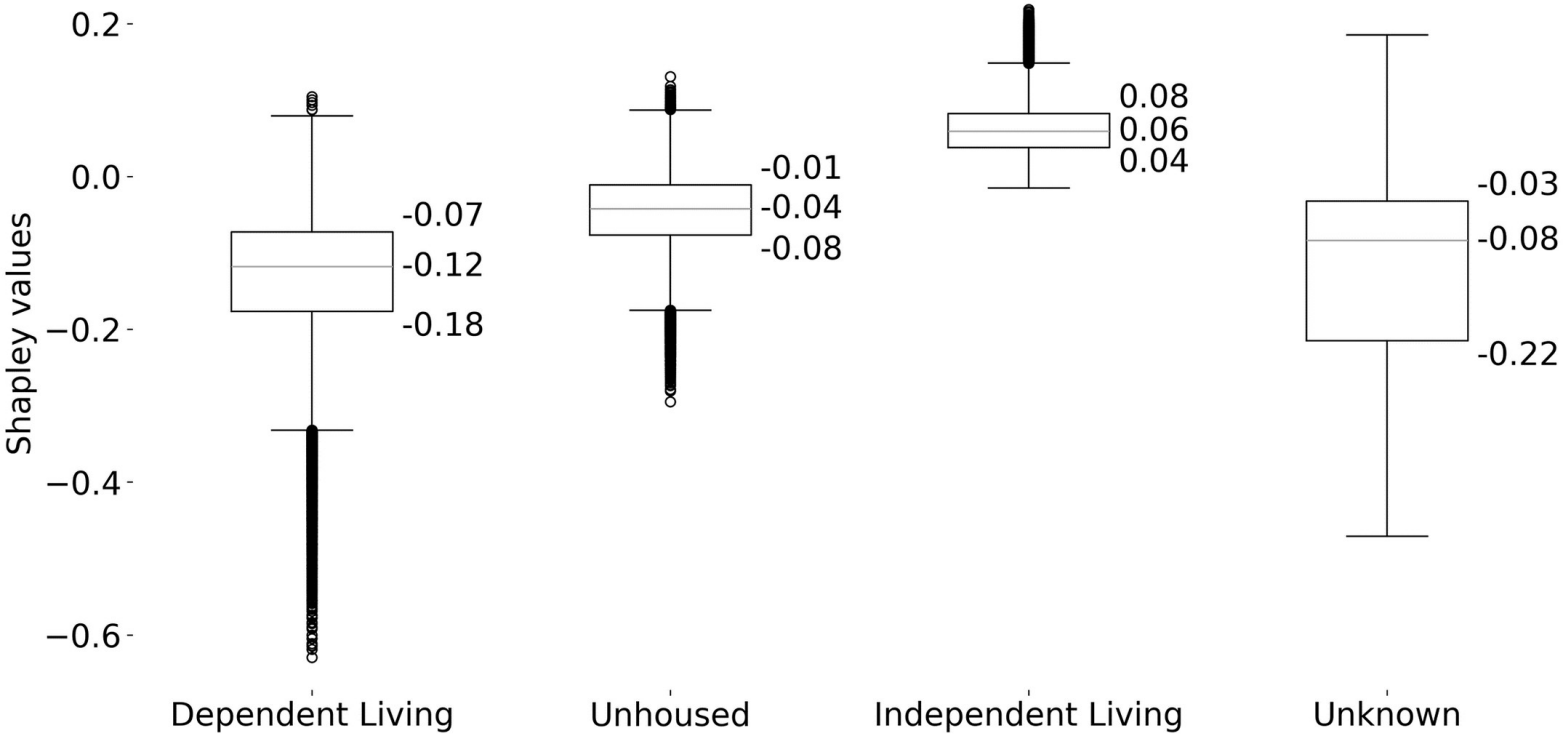
Shapley values associated with a patient’s living arrangement. Independent living most often predicts MOUD access while unhoused and dependent living most often predict no MOUD access. This box plot shows the distribution of Shapley values for individual admissions by housing status, or in other words, how much does each admission’s housing status influence a positive or negative prediction of MOUD access. The top and bottom of each box represent the interquartile range of Shapley values, and the line in between represents the median.

Fig 3 separates all admissions by their living arrangement and shows the distribution of Shapley values associated with the living arrangement of each subgroup. Admissions with an independent living housing status have the highest median Shapley value at 0.06 and an interquartile range of 0.04 to 0.08, indicating that the subgroup is statistically more likely to access MOUD. Conversely, we have unhoused admissions with a median Shapley value of -0.04 and an interquartile range of -0.01 to -0.08. This wider range and negative value indicate that a lack of secure housing is associated with greater uncertainty as well as lower rates of accessing MOUD. This is even worse for admissions of those in dependent living arrangements whose median Shapley value is lower (-0.12) and with a wider range (-0.07 to -0.18) compared to unhoused admissions. Finally, a majority of admissions with an “Unknown” living arrangement have a Shapley value of -0.07 or lower, suggesting that missing living arrangement data is most often predictive of no MOUD treatment for opioid addiction. Due to this predictive power, we decided to not drop the “Unknown” living status values.

Overall, these results demonstrate that living arrangement can generate either a positive or negative signal that predicts MOUD access. The differences highlight the disparities in MOUD access among those who do not live independently.

### Access disparities by housing status

Recall from Table 1, that the most important feature within our dataset is the service setting (SERVICES variable), contributing to nearly a quarter of the model’s predictive capacity. This feature is meaningful for clinicians because 1) it describes other aspects of care available to the patient (e.g., *detoxification*, *24-hour service*, *hospital inpatient*), 2) presents an intervention opportunity for improving MOUD access by directing patients with OUD to the appropriate facility, and 3) suggests that service setting is associated with the disparity of care between patients who live independently and those experiencing homelessness. We use these insights from the model’s feature importance to take a fresh look at the relation of housing and service setting with MOUD access. The rest of this section exclusively uses rates of treatment from the original dataset.

There is a high correlation between treatment facility type and MOUD treatment, with unhoused patients being more likely to be admitted to a treatment facility with low rates of MOUD. Table 2 shows rates of MOUD access across such service settings, broken down by patient housing status. Below the MOUD access rates for unhoused admissions is a value in parentheses that shows the percent difference between an access rate for unhoused admissions and an access rate for independent living admissions. We also include percent differences between dependent living admissions and independent living admissions. The final column lists the share of observations coming from each of these service settings.

**Table 2:** MOUD treatment rates by service setting and living arrangement.

Type of treatment service setting (SERVICES)	Unhoused MOUD treatment rate	Dependent MOUD treatment rate	Independent MOUD treatment rate	Share of opioid addiction admissions
Ambulatory, detoxification	63.70%	30.33%	47.19%	0.95%
	(34.97%****)	(-35.73%****)		
Ambulatory, intensive outpatient	18.76%	16.41%	27.87%	11.01%
	(-32.69%****)	(-41.12%****)		
Ambulatory, non-intensive outpatient	51.32%	63.48%	63.60%	50.17%
	(-19.31%****)	(-0.19%)		
Detox, 24-hour, free-standing residential	19.41%	12.57%	13.87%	18.53%
	(39.98%****)	(-9.33%****)		
Detox, 24-hour, hospital inpatient	3.03%	1.04%	0.80%	2.38%
	(276.71%****)	(29.58%)		
Rehab/residential, hospital (non-detox)	15.38%	64.13%	30.35%	0.14%
	(-49.31%****)	(111.29%****)		
Rehab/residential, short term (more than 30 days)	28.72%	25.58%	29.99%	6.74%
	(-4.24%**)	(-14.69%****)		
Rehab/residential, short term (30 days or fewer)	14.13%	26.37%	25.34%	10.09%
	(-44.24%****)	(4.06%**)		

Values in parentheses are percent differences from independent living admissions with statistical significance shown at the 0.1 (*), 0.05 (**), 0.01 (***), and 0.001 (****) levels. Over half of all admitted patients with OUD go to the Ambulatory, non-intensive outpatient setting, yet those unhoused patients are less likely to receive MOUD while there than housed patients. Other service settings provide higher or lower MOUD access to housed patients than their unhoused counterparts.

The first insight from Table 2 is that for most service settings, unhoused admissions receive MOUD at lower rates than independent living admissions. Even though patients of all housing types are admitted to all facility types, patients are not receiving equal treatment within each service setting type. For example, over half of all admissions are at *Ambulatory*, *non-intensive outpatient*; however, an independent living admission receives MOUD 63.60% of the time whereas an unhoused admission receives MOUD 51.32% of the time. This is 19.31% less and statistically significant at the 0.001 level.

We also find in Table 2 that there are three service settings where unhoused admissions receive MOUD at higher rates than independent living admissions: *Ambulatory*, *detoxification* at a 34.97% higher rate; *Detox*, *24-hour*, *free-standing residential* at a 39.98% higher rate; and *Detox*, *24-hour*, *hospital inpatient* at a 276.71% higher rate; all differences are statistically significant at the 0.001 level. Although comprising a small percent of our population, it does appear that the *Detox*, *24-hour*, *hospital inpatient* service setting provides greater access to MOUD for patients experiencing homelessness.

In one final observation about Table 2, we see that rates of MOUD access generally vary more by service setting types than by housing status. For example, among the unhoused, MOUD access is as low as 3.03% for *Detox*, *24-hour*, *hospital inpatient* and as high as 63.70% for *Ambulatory*, *detoxification* (a 60.67 percentage point difference). Conversely, among admissions to *Ambulatory*, *detoxification* facilities, the largest difference in MOUD treatment rate between unhoused (63.70%) and independent living (47.19%) patients is only 16.51 percentage points. This insight suggests the problem may occur further upstream and before a patient even arrives at a treatment facility.

In fact, we find a wide difference in the service setting that patients with OUD end up going to, and this has a significant impact on whether a patient receives MOUD. To visualize this difference, we plot the path a patient takes to receive treatment as being mediated first by the type of service setting they enter. We break this down in a pair of Sankey diagrams where the thickness of each flow path is determined by the share of admissions within a given housing status.

**Fig 4:**
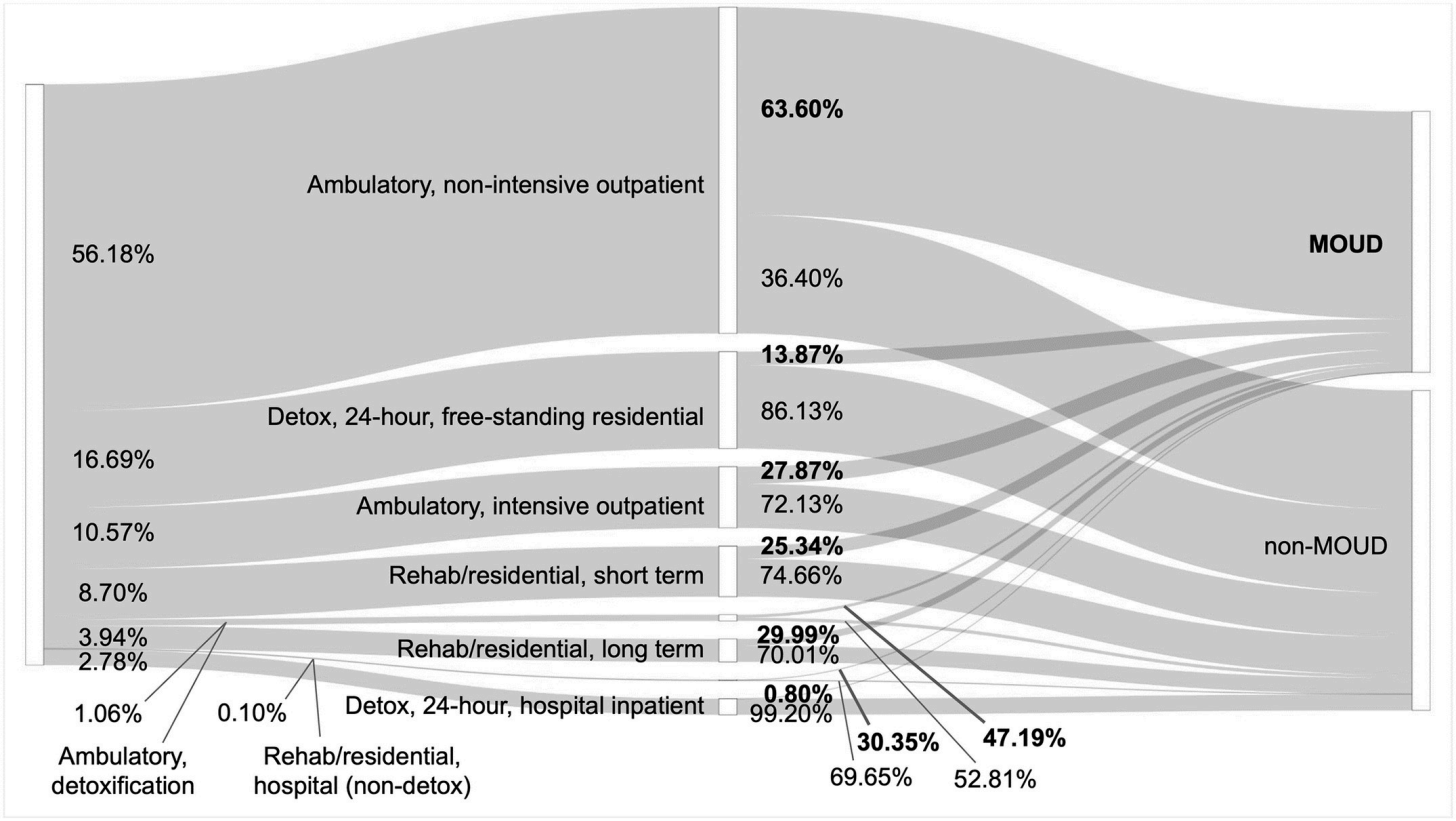
The path of independent living admissions by service setting and treatment outcome. This Sankey chart shows that most independent living patients with OUD are admitted to the *Ambulatory*, *non-intensive outpatient* service setting where they are more likely to receive MOUD. Other service settings are associated with lower rates of MOUD (e.g., *Ambulatory*, *intensive outpatient*; *Detox*, *24-hour*, *free-standing residential*; *Rehab/residential*, *short term*), but independent living patients are less likely to go to those facilities.

For example, Fig 4 shows that most independent living admissions enter *Ambulatory non-intensive outpatient* service settings (56.18%), and from there, a majority of those patients receive MOUD (63.60%). It appears that this facility type is rather effective at providing MOUD and that patients who live independently are disproportionately able to access such a service setting.

**Fig 5:**
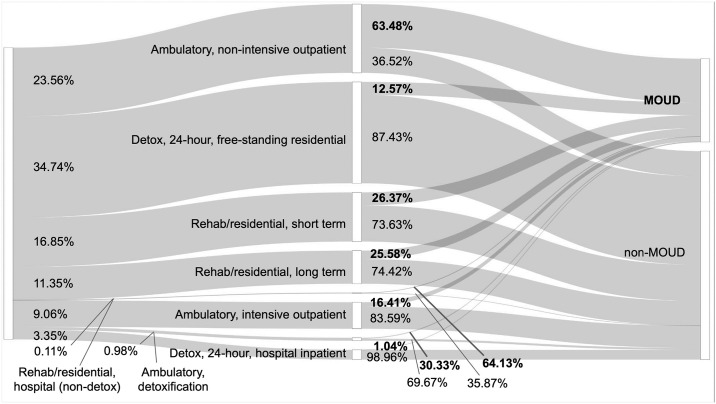
The path of unhoused admissions by service setting and treatment outcome. This Sankey chart shows that a plurality of unhoused patients with OUD are admitted to the *Detox*, *24-hour*, *free-standing residential* service setting where they are less likely to receive MOUD. Comparing this with Fig 4 shows that unhoused patients are less likely than independent living patients to go to the types of facilities with higher rates of MOUD access.

Next is Fig 5, showing the path of unhoused patients as they are admitted to various service settings and whether they receive MOUD. There is a stark difference in the distribution of admissions between these two charts. In Fig 5, the largest group of unhoused admissions enter *Detox*, *24-hour*, *free-standing residential* facilities (34.74%), and from there, the majority of these admissions do not receive MOUD (87.43%). These patients would perhaps be better served by going to other service settings, such as the aforementioned *Ambulatory non-intensive outpatient* facilities where MOUD treatment rates are higher at 63.48%; however, we see that only 23.56% of unhoused admissions end up there. Together these charts show that the disparity in MOUD access among unhoused and independent living patients is also a disparity in service setting access.

Lastly, we want to quantify the difference between the present state and a counterfactual reality where we imagine that unhoused patients have equal access to treatment facilities. In this “what-if scenario,” we consider a single patient affected by homelessness being randomly assigned to a service setting at the same frequency of an independent living patient, but once there, is treated for MOUD at the same rate of an unhoused patient.

Specifically, suppose unhoused patients entered *Detox*, *24-hour*, *free-standing residential* facilities only 16.69% of the time (the rate of independent living patients) rather than 34.74% of the time (the rate for unhoused patients); but once there, still received MOUD treatment 12.57% of the time (the rate for unhoused patients). Put differently, this is as if unhoused patients entered service settings in a manner like the left half of Fig 4 (mirroring the trend of independent living patients) but still received MOUD like the right half of Fig 5.

In this what-if scenario, 43.20% of unhoused admissions would receive MOUD (scaled up from the current rate of 28.56%). We benchmark this against the current MOUD treatment rate for independent admissions of 44.92%. In other words, 89.50% of the current access disparity between these two patient types would be eliminated simply by unhoused patients entering the same service setting as independent living patients. (Note: The present disparity in MOUD access between independent and unhoused admissions is 44.92%–28.56% = 16.36 points and the disparity in the counterfactual is 44.92%–43.20% = 1.72 points. This is a difference of (16.36–1.72) / 16.36 = 89.50%.)

This thought experiment, however, crucially assumes unhoused patients have equal access to treatment facilities as their housed counterparts. There are several limitations within the source dataset from SAMHSA that preclude us from testing this assumption or measuring the geographic distribution of facilities and patients of various living statuses. First, some pairs of demographically similar admissions are swapped to different locations to preserve patient privacy. This adds negligible bias on aggregate, but makes it unclear whether a specific admission at one facility type is truly at the listed geography. Second, the lowest level of geographic granularity is at the metropolitan or micropolitan statistical area (MSA) and is too large to make inferences about accessibility. As an extreme example, the New York City MSA includes approximately 20 million residents living in parts of New York, New Jersey, Connecticut, and Pennsylvania. Finally, MSAs with fewer than 100,000 residents are excluded entirely which is possibly why 43.18% of admissions have no known MSA.

## Discussion

MOUD is the preferred treatment for patients with OUD [26], yet our study shows that only 44.92% of independent living admissions and 28.56% of unhoused admissions have access to MOUD. We hypothesize the discrepancy in access to these needed medications occurs at the service setting level, and we therefore recommend increased access to MOUD at non-traditional service settings. There is some research in this area, such as one study that found emergency department patients who received buprenorphine treatment for OUD were twice as likely to still be receiving OUD treatment 30 days later [27]. Similarly, Austin and colleagues propose providing MOUD in the primary care setting [28].

Approaches for unhoused patients with a substance use disorder include a rapid housing model; additional concurrent interventions, including the incorporation of access to service settings that offer MOUD, may play a role in the success of such strategies [4, 7].

Our research suggests that increasing treatment options for OUD patients will improve outcomes. In spite of this, Rosenberg and colleagues find in a meta analysis of the addiction literature that caregivers’ attitudes toward OUD treatment varies widely by service setting and geography [29]. Educational outreach programs can increase MOUD prescriptions, as reported in a study in Michigan that focused on barriers to access by different communities [30].

There are several limitations of our study in relation to our data and methodology. First, the TEDS-A dataset is comprised of admissions from publicly-funded facilities reporting to individual states within a mix of mandatory and optional standards; additionally, due to inconsistent reporting, privately-funded admissions are not recorded in TEDS-A, which we anticipate would have a larger skew on treatment rates for housed patients than unhoused patients. Second, the TEDS-A dataset is shown at the admission level; as such, patients with multiple admissions within the same calendar year are reported multiple times. Third, although we recommend increasing access for unhoused patients to receive treatment at MOUD-offering service settings, we do not assess the geographic proximity of these facilities to this patient population. Finally, Shapley values are always calculated in relation to the features used by a model. As a result, the feature importance reported will inevitably change with each feature added or removed. While we took steps to include all available data, our results should be viewed as relative importance that is compared against the other included features.

There are various ways to expand on this research. Future work might further explore how OUD patients with *dependent* living arrangements (e.g., group homes, boarding schools, correctional facilities, etc.) receive MOUD; as shown in Fig 3, dependent living patients were more strongly associated with not having access to MOUD than even unhoused patients. Subsequent work might utilize a similar methodology to predict *where* a patient with OUD is admitted and which factors are more associated with each facility; this will better elucidate how upstream factors beyond housing status impact MOUD access. Finally, an exploration of how unhoused patients with OUD fared during and after the pandemic may yield meaningful insights; relatedly, our study used the 2019 TEDS-A to avoid potential confounders introduced by the COVID-19 pandemic in later TEDS-A releases. Tormohlen and colleagues show that OUD treatment delivery experienced fewer disruptions than anticipated during the pandemic, due to the rapid expansion of telehealth and other virtual services from 2020 onwards [31]; still, although telehealth can increase OUD treatment retention [32], unhoused patients are less likely to have the necessary technology to receive such care.

## Conclusion

Overdose deaths in the United States are on the rise and do not show signs of stopping, especially with the proliferation of synthetic opioids such as fentanyl and the rise in polysubstance use. Despite the efficacy of MOUD treatment, disparities in access persist. Previous literature reports social determinants of health closely influencing MOUD access [5, 16, 33]; in particular, unhoused patients are more likely to suffer from OUD, yet are less likely to receive MOUD treatment [6, 19].

Our results support these observations and offer a complementary, quantitative association with inequitable access in care for the unhoused by using modern machine learning methods to explain the discrepancy. We hypothesize that housing status has a small direct effect on MOUD access but has a large indirect effect, attributable to the observed tendencies of unhoused patients to go to treatment centers with lower MOUD access, and of housed patients to go to treatment centers with higher MOUD access (comparing Figs 4 and 5). We demonstrate that the disparity in MOUD access among unhoused patients is associated with their access to certain service settings, rather than how they are treated once admitted. Consequently, this vulnerable population may be best served by future policy and research efforts focused on increasing access to MOUD-administering service settings.

## Supporting information

S1 Appendix MOUD access rates by living arrangement. (PDF)

S2 Appendix Admission counts and frequencies by subgroup. (PDF)

S3 Appendix Full feature list. (PDF)

S1 Data (XLSX)
