# Supplementary material for: Factors predicting access to medications for opioid use disorder for housed and unhoused patients: A machine learning approach
Source: PLoS One. 2024 Sep 27;19(9):e0308791. doi: 10.1371/journal.pone.0308791 (PMC11433129; doi:10.1371/journal.pone.0308791)
Supplement: S1 Appendix — (PDF) [file pone.0308791.s001.pdf]

S1 Appendix - MOUD Access Rates by Living Arrangement

Group	Subgroup	Total MOUD Treatment Rate	Unhoused MOUD Treatment Rate	Dependent MOUD Treatment Rate	Independent MOUD Treatment Rate	% Difference - Unhoused from Independent Living
ALL	ALL	40.40%	28.56%	34.54%	44.92%	-36.42%****
AGE	Age12To14	61.02%		10.26%	51.35%	
AGE	Age15To17	23.34%	20.00%	5.61%	17.73%	12.78%
AGE	Age18To20	30.08%	21.02%	25.58%	32.84%	-36.01%****
AGE	Age21To24	32.29%	23.87%	29.70%	34.85%	-31.52%****
AGE	Age25To29	35.63%	25.99%	32.39%	38.80%	-33.01%****
AGE	Age30To34	39.38%	28.99%	34.60%	43.26%	-33.00%****
AGE	Age35To39	41.13%	29.67%	36.45%	45.27%	-34.45%****
AGE	Age40To44	42.16%	30.21%	37.76%	46.69%	-35.30%****
AGE	Age45To49	42.58%	28.17%	34.70%	48.82%	-42.31%****
AGE	Age50To54	45.38%	28.48%	35.17%	53.01%	-46.28%****
AGE	Age55To64	49.95%	32.29%	39.70%	56.24%	-42.59%****
AGE	Age65Plus	59.02%	36.24%	49.89%	64.18%	-43.53%****
ALCDRUG	AlcoholAndDrugs	30.13%	23.86%	30.14%	33.05%	-27.80%****
ALCDRUG	OtherDrugs	41.89%	29.51%	35.14%	46.58%	-36.65%****
ALCFLG	NotReported	41.89%	29.51%	35.14%	46.58%	-36.65%****
ALCFLG	Reported	30.13%	23.86%	30.14%	33.05%	-27.80%****
AMPHFLG	NotReported	40.45%	28.57%	34.58%	44.99%	-36.49%****
AMPHFLG	Reported	34.20%	27.12%	31.18%	37.29%	-27.27%****
ARRESTS	0Arrest	42.40%	31.72%	34.55%	48.30%	-34.32%****
ARRESTS	1Arrest	37.64%	29.87%	36.32%	37.44%	-20.20%****
ARRESTS	2PlusArrest	31.73%	30.60%	30.80%	35.70%	-14.29%***
ARRESTS	Unknown	21.80%	1.89%	20.29%	11.86%	-84.09%****
BARBFLG	NotReported	40.41%	28.57%	34.55%	44.93%	-36.42%****
BARBFLG	Reported	24.18%	13.46%	27.71%	25.13%	-46.43%*
BENZFLG	NotReported	41.11%	28.96%	34.57%	45.84%	-36.82%****
BENZFLG	Reported	32.13%	24.84%	34.12%	34.50%	-28.00%****
CBSA2010	*Excluded due to large number of subgroups (272)*					
COKEFLG	NotReported	40.64%	27.85%	33.75%	44.97%	-38.07%****
COKEFLG	Reported	39.67%	29.97%	36.72%	44.77%	-33.04%****
DAYWAIT	0DaysWait	42.42%	26.00%	29.76%	49.60%	-47.58%****
DAYWAIT	15To30DaysWait	36.83%	28.26%	26.06%	42.67%	-33.76%****
DAYWAIT	1To7DaysWait	45.14%	30.57%	40.74%	50.59%	-39.58%****
DAYWAIT	31PlusDaysWait	34.91%	27.93%	22.10%	39.69%	-29.63%****
DAYWAIT	8To14DaysWait	41.24%	27.92%	34.16%	46.79%	-40.32%****
DAYWAIT	Unknown	37.07%	30.62%	41.85%	39.26%	-22.01%****
DIVISION	EastNorthCentral	36.16%	37.20%	20.02%	43.96%	-15.36%****
DIVISION	EastSouthCentral	16.49%	5.35%	20.94%	18.04%	-70.33%****
DIVISION	MiddleAtlantic	50.98%	38.20%	52.16%	53.78%	-28.98%****
DIVISION	Mountain	32.92%	28.54%	23.05%	39.39%	-27.54%****
DIVISION	NewEngland	35.36%	24.46%	39.81%	38.53%	-36.50%****
DIVISION	Pacific	58.14%	34.40%	54.51%	70.89%	-51.47%****
DIVISION	SouthAtlantic	37.85%	14.17%	21.35%	41.23%	-65.62%****

Statistical significance shown at the 0.1 (*), 0.05 (**), 0.01 (***), and 0.001 (****) levels.

Group	Subgroup	Total MOUD Treatment Rate	Unhoused MOUD Treatment Rate	Dependent MOUD Treatment Rate	Independent MOUD Treatment Rate	% Difference - Unhoused from Independent Living
DIVISION	USTerritories	26.15%	15.83%	49.32%	25.88%	-38.85%**
DIVISION	WestNorthCentral	34.90%	34.78%	36.77%	34.52%	0.76%
DIVISION	WestSouthCentral	18.32%	7.68%	19.85%	21.22%	-63.80%****
DSMCRIT	ADDOrBehaviorDisorder	31.58%	16.67%	66.67%	32.14%	-48.15%
DSMCRIT	AlcoholAbuse	19.24%	19.48%	13.13%	23.09%	-15.63%
DSMCRIT	AlcoholDependence	20.14%	20.00%	18.53%	21.49%	-6.92%
DSMCRIT	AlcoholDisorder	25.24%	16.67%	50.00%	25.00%	-33.33%
DSMCRIT	AlcoholIntoxication	2.99%	0.00%	0.00%	4.76%	-100.00%
DSMCRIT	AnxietyDisorder	30.64%	14.63%	26.56%	34.53%	-57.62%**
DSMCRIT	BipolarDisorder	21.58%	15.91%	8.93%	27.70%	-42.57%
DSMCRIT	CannabisAbuse	14.20%	14.52%	10.00%	15.82%	-8.27%
DSMCRIT	CannabisDependence	22.14%	27.33%	15.40%	24.65%	10.84%
DSMCRIT	CocaineAbuse	18.72%	23.08%	13.92%	19.15%	20.51%
DSMCRIT	CocaineDependence	22.16%	24.37%	17.28%	24.19%	0.74%
DSMCRIT	DepressiveDisorder	22.53%	19.78%	28.97%	22.63%	-12.59%
DSMCRIT	OpioidAbuse	26.92%	24.96%	34.92%	27.94%	-10.68%**
DSMCRIT	OpioidDependence	42.26%	30.32%	30.47%	47.00%	-35.48%****
DSMCRIT	OtherMentalHealthCondition	47.97%	33.91%	66.27%	50.14%	-32.36%****
DSMCRIT	OtherSubstanceAbuse	12.43%	13.04%	14.52%	13.72%	-4.95%
DSMCRIT	OtherSubstanceDependence	15.69%	20.92%	13.54%	16.92%	23.64%***
DSMCRIT	SchizopheniaOrPsychDisorder	13.86%	5.26%	5.56%	17.46%	-69.86%
DSMCRIT	SubstanceDisorder	13.33%	5.26%	13.79%	15.70%	-66.47%****
DSMCRIT	Unknown	39.92%	27.21%	42.04%	45.14%	-39.73%****
EDUC	1To3yCollege	39.74%	30.79%	37.18%	42.72%	-27.94%****
EDUC	4yCollegePlus	40.13%	24.72%	31.27%	45.04%	-45.13%****
EDUC	Grade12OrGED	39.60%	27.66%	33.56%	44.65%	-38.05%****
EDUC	Grade8OrLess	35.74%	26.26%	36.75%	39.95%	-34.27%****
EDUC	Grade9To11	41.72%	30.71%	33.91%	47.55%	-35.42%****
EDUC	Unknown	49.63%	28.62%	47.71%	57.59%	-50.30%****
EMPLOY_DETNLF	FullTime	42.23%	30.68%	32.37%	44.43%	-30.95%****
EMPLOY_DETNLF	Homemaker	45.55%	27.22%	34.84%	47.45%	-42.64%****
EMPLOY_DETNLF	InstitutionResident	29.15%	26.11%	29.91%	26.69%	-2.18%
EMPLOY_DETNLF	OtherNotInLaborForce	35.46%	25.58%	36.33%	38.37%	-33.33%****
EMPLOY_DETNLF	PartTime	46.51%	37.03%	40.72%	48.56%	-23.75%****
EMPLOY_DETNLF	RetiredOrDisabled	48.98%	34.09%	43.93%	52.34%	-34.87%****
EMPLOY_DETNLF	Student	31.45%	25.45%	20.30%	36.37%	-30.01%**
EMPLOY_DETNLF	Unemployed	37.21%	28.72%	30.82%	40.81%	-29.63%****
EMPLOY_DETNLF	Unknown	57.36%	32.59%	41.74%	73.82%	-55.86%****
EMPLOY_DETNLF	UnknownNotInLaborForce	55.77%	33.62%	51.59%	70.81%	-52.51%****
ETHNIC	CubanOrOther	37.11%	27.89%	35.89%	40.69%	-31.45%****
ETHNIC	Mexican	52.22%	34.07%	41.41%	62.01%	-45.07%****
ETHNIC	NonHispanic	39.38%	28.09%	33.56%	44.48%	-36.84%****
ETHNIC	PuertoRican	43.62%	31.99%	47.25%	47.82%	-33.09%****
ETHNIC	Unknown	55.68%	31.87%	45.68%	37.30%	-14.56%****
ETHNIC	UnspecifiedHispanic	38.71%	24.39%	30.88%	43.67%	-44.13%****

Statistical significance shown at the 0.1 (*), 0.05 (**), 0.01 (***), and 0.001 (****) levels.

Group	Subgroup	Total MOUD Treatment Rate	Unhoused MOUD Treatment Rate	Dependent MOUD Treatment Rate	Independent MOUD Treatment Rate	% Difference - Unhoused from Independent Living
FREQ_ATND_SELF_HELP	1To3TimesPastMonth	36.79%	21.31%	28.47%	44.32%	-51.93%****
FREQ_ATND_SELF_HELP	4To7TimesPastMonth	33.87%	25.38%	28.87%	38.89%	-34.74%****
FREQ_ATND_SELF_HELP	8To30TimesPastMonth	30.22%	22.08%	29.38%	34.30%	-35.63%****
FREQ_ATND_SELF_HELP	NoAttendance	42.62%	32.27%	35.10%	48.22%	-33.06%****
FREQ_ATND_SELF_HELP	SomeAttendance	71.60%	59.26%	64.17%	66.89%	-11.41%****
FREQ_ATND_SELF_HELP	Unknown	23.43%	11.10%	25.46%	25.77%	-56.95%****
FREQ1	DailyUse	40.00%	27.41%	34.60%	45.05%	-39.15%****
FREQ1	NoUsePastMonth	41.03%	35.54%	34.25%	45.98%	-22.70%****
FREQ1	SomeUse	38.46%	28.10%	35.29%	42.83%	-34.40%****
FREQ1	Unknown	62.85%	20.11%	22.27%	47.22%	-57.41%****
FREQ2	DailyUse	32.06%	24.45%	30.81%	36.31%	-32.66%****
FREQ2	NoUsePastMonth	37.99%	33.83%	32.87%	42.52%	-20.43%****
FREQ2	SomeUse	40.17%	31.63%	36.20%	44.46%	-28.86%****
FREQ2	Unknown	47.24%	30.16%	37.35%	51.25%	-41.15%****
FREQ3	DailyUse	29.83%	22.96%	26.97%	34.12%	-32.70%****
FREQ3	NoUsePastMonth	38.32%	37.08%	33.54%	42.04%	-11.79%****
FREQ3	SomeUse	37.80%	31.93%	34.97%	40.74%	-21.62%****
FREQ3	Unknown	42.29%	28.65%	35.51%	47.02%	-39.06%****
FRSTUSE1	11yLess	34.91%	23.79%	28.59%	44.12%	-46.09%****
FRSTUSE1	12To14y	38.00%	26.27%	31.70%	45.81%	-42.66%****
FRSTUSE1	15To17y	39.26%	29.14%	33.41%	44.86%	-35.04%****
FRSTUSE1	18To20y	39.75%	28.48%	35.56%	44.72%	-36.33%****
FRSTUSE1	21To24y	37.67%	28.18%	33.52%	42.11%	-33.07%****
FRSTUSE1	25To29y	38.96%	28.37%	34.85%	43.62%	-34.95%****
FRSTUSE1	30yPlus	41.23%	30.26%	35.99%	45.88%	-34.04%****
FRSTUSE1	Unknown	70.27%	23.93%	48.88%	66.01%	-63.74%****
FRSTUSE2	11yLess	31.93%	23.05%	27.56%	37.94%	-39.24%****
FRSTUSE2	12To14y	35.23%	26.96%	34.21%	39.19%	-31.21%****
FRSTUSE2	15To17y	36.47%	28.61%	35.56%	40.14%	-28.73%****
FRSTUSE2	18To20y	37.63%	29.02%	37.21%	41.34%	-29.80%****
FRSTUSE2	21To24y	37.21%	29.12%	35.45%	41.30%	-29.49%****
FRSTUSE2	25To29y	37.52%	28.54%	37.62%	41.52%	-31.27%****
FRSTUSE2	30yPlus	40.20%	30.89%	39.26%	44.10%	-29.95%****
FRSTUSE2	Unknown	45.40%	28.69%	32.59%	50.47%	-43.15%****
FRSTUSE3	11yLess	32.88%	25.40%	29.10%	38.13%	-33.38%****
FRSTUSE3	12To14y	35.34%	28.93%	32.58%	39.05%	-25.91%****
FRSTUSE3	15To17y	35.96%	29.95%	33.71%	39.17%	-23.54%****
FRSTUSE3	18To20y	36.07%	29.25%	35.57%	38.84%	-24.68%****
FRSTUSE3	21To24y	36.61%	27.84%	38.49%	39.50%	-29.52%****
FRSTUSE3	25To29y	36.36%	29.14%	39.83%	38.71%	-24.72%****
FRSTUSE3	30yPlus	37.64%	28.80%	41.05%	40.40%	-28.72%****
FRSTUSE3	Unknown	41.96%	28.44%	34.51%	46.88%	-39.33%****
GENDER	Female	43.39%	31.19%	36.28%	47.73%	-34.66%****
GENDER	Male	38.59%	27.44%	33.52%	43.16%	-36.42%****
GENDER	Unknown	84.73%	45.95%	53.33%	57.28%	-19.79%

Statistical significance shown at the 0.1 (*), 0.05 (**), 0.01 (***), and 0.001 (****) levels.

Group	Subgroup	Total MOUD Treatment Rate	Unhoused MOUD Treatment Rate	Dependent MOUD Treatment Rate	Independent MOUD Treatment Rate	% Difference - Unhoused from Independent Living
HALLFLG	NotReported	40.42%	28.56%	34.58%	44.93%	-36.44%****
HALLFLG	Reported	31.02%	28.43%	21.05%	38.29%	-25.76%**
HERFLG	NotReported	40.77%	26.24%	32.36%	40.09%	-34.55%****
HERFLG	Reported	40.31%	28.78%	34.95%	46.31%	-37.86%****
HLTHINS	Medicaid	41.63%	23.60%	29.09%	49.58%	-52.41%****
HLTHINS	MedicareOrOther	37.63%	23.58%	37.18%	39.20%	-39.86%****
HLTHINS	None	23.19%	13.60%	20.08%	27.53%	-50.62%****
HLTHINS	PrivateInsurance	39.38%	26.83%	22.01%	42.52%	-36.90%****
HLTHINS	Unknown	44.15%	35.67%	45.48%	46.01%	-22.46%****
IDU	IDU	39.44%	29.61%	35.40%	45.00%	-34.19%****
IDU	NoIDU	41.35%	26.91%	33.52%	44.86%	-40.00%****
INHFLG	NotReported	40.40%	28.56%	34.54%	44.92%	-36.43%****
INHFLG	Reported	35.22%	33.33%	38.30%	36.59%	-8.89%
LIVARAG	DependLiving	34.54%				
LIVARAG	Unhoused	28.56%				
LIVARAG	IndependentLiving	44.92%				
LIVARAG	Unknown	41.41%				
MARFLG	NotReported	41.32%	28.41%	35.70%	46.01%	-38.24%****
MARFLG	Reported	35.74%	29.43%	28.77%	39.81%	-26.08%****
MARSTAT	DivorcedOrWidowed	42.40%	32.61%	31.18%	47.96%	-32.01%****
MARSTAT	NeverMarried	40.95%	30.62%	32.82%	46.31%	-33.87%****
MARSTAT	NowMarried	47.95%	35.39%	33.57%	50.60%	-30.07%****
MARSTAT	Separated	39.27%	31.77%	31.30%	44.64%	-28.83%****
MARSTAT	Unknown	34.66%	20.27%	43.20%	35.00%	-42.08%****
METHFLG	NotReported	40.34%	28.58%	34.52%	44.85%	-36.28%****
METHFLG	Reported	47.27%	25.83%	37.52%	51.59%	-49.92%****
METHUSE	0	0.00%	0.00%	0.00%	0.00%	
METHUSE	1	100.00%	100.00%	100.00%	100.00%	0.00%
MTHAMFLG	NotReported	42.03%	28.99%	36.13%	46.18%	-37.23%****
MTHAMFLG	Reported	29.62%	26.62%	27.52%	33.76%	-21.14%****
NOPRIOR	0PriorTreatments	44.55%	29.49%	37.24%	49.14%	-39.98%****
NOPRIOR	1PriorTreatments	43.41%	30.49%	30.01%	48.13%	-36.64%****
NOPRIOR	2PriorTreatments	44.16%	35.00%	31.01%	51.49%	-32.03%****
NOPRIOR	3PriorTreatments	44.64%	34.14%	35.52%	51.55%	-33.77%****
NOPRIOR	4PriorTreatments	43.30%	35.63%	34.61%	50.26%	-29.11%****
NOPRIOR	5PlusPriorTreatments	41.54%	32.87%	39.72%	45.95%	-28.47%****
NOPRIOR	Unknown	10.19%	2.96%	15.01%	11.18%	-73.51%****
OPSYNFLG	NotReported	39.95%	28.43%	34.62%	46.10%	-38.32%****
OPSYNFLG	Reported	41.64%	29.39%	34.28%	42.13%	-30.24%****
OTCFLG	NotReported	40.40%	28.56%	34.55%	44.92%	-36.42%****
OTCFLG	Reported	40.10%	35.00%	26.09%	48.75%	-28.21%
OTHERFLG	NotReported	40.48%	28.60%	34.59%	45.03%	-36.48%****
OTHERFLG	Reported	35.56%	26.20%	31.55%	39.27%	-33.27%****
PCPFLG	NotReported	40.42%	28.58%	34.56%	44.94%	-36.41%****
PCPFLG	Reported	28.36%	22.62%	22.07%	33.27%	-32.01%***

Statistical significance shown at the 0.1 (*), 0.05 (**), 0.01 (***), and 0.001 (****) levels.

Group	Subgroup	Total MOUD Treatment Rate	Unhoused MOUD Treatment Rate	Dependent MOUD Treatment Rate	Independent MOUD Treatment Rate	% Difference - Unhoused from Independent Living
PREG	NotPregnant	43.99%	32.79%	35.75%	49.59%	-33.88%****
PREG	Pregnant	55.37%	45.70%	51.79%	58.81%	-22.28%****
PREG	Unknown	38.26%	26.76%	33.44%	42.05%	-36.37%****
PRIMINC	None	30.22%	24.29%	26.41%	34.64%	-29.89%****
PRIMINC	Other	43.82%	28.30%	41.58%	47.91%	-40.93%****
PRIMINC	PublicAssist	49.72%	34.14%	51.03%	56.41%	-39.48%****
PRIMINC	RetireOrDisable	48.42%	26.18%	38.90%	54.63%	-52.08%****
PRIMINC	Unknown	42.11%	32.23%	36.59%	45.40%	-29.01%****
PRIMINC	Wages	43.57%	29.78%	27.92%	47.07%	-36.73%****
PRIMPAY	Medicaid	45.29%	26.67%	28.93%	54.11%	-50.71%****
PRIMPAY	Medicare	46.84%	29.80%	38.29%	57.96%	-48.59%****
PRIMPAY	NoCharge	15.65%	13.38%	18.04%	15.79%	-15.27%*
PRIMPAY	Other	30.60%	21.41%	18.22%	31.54%	-32.11%****
PRIMPAY	OtherGovtPayment	32.57%	15.04%	29.36%	35.85%	-58.04%****
PRIMPAY	PrivateInsurance	38.90%	32.99%	24.31%	41.91%	-21.28%****
PRIMPAY	SelfPay	48.94%	24.92%	33.64%	53.59%	-53.49%****
PRIMPAY	Unknown	39.56%	31.18%	40.49%	42.22%	-26.14%****
PSOURCE_DETCRIM	Adjudication	15.30%	12.71%	10.99%	18.78%	-32.33%**
PSOURCE_DETCRIM	Court	27.99%	25.62%	22.04%	31.27%	-18.08%****
PSOURCE_DETCRIM	DUI	33.66%	22.22%	11.45%	36.90%	-39.77%
PSOURCE_DETCRIM	DiversionaryProgram	27.23%	29.63%	21.18%	30.52%	-2.91%
PSOURCE_DETCRIM	DrugCareProvider	41.19%	32.34%	37.86%	46.51%	-30.47%****
PSOURCE_DETCRIM	Employer	17.30%	5.43%	10.89%	24.05%	-77.40%****
PSOURCE_DETCRIM	Individual	46.12%	29.10%	44.03%	51.54%	-43.54%****
PSOURCE_DETCRIM	Other	9.21%	6.84%	8.23%	10.43%	-34.40%***
PSOURCE_DETCRIM	OtherHealthCareProvider	33.61%	29.47%	28.22%	37.21%	-20.78%****
PSOURCE_DETCRIM	OtherLegalEntity	43.09%	50.13%	38.30%	43.90%	14.21%**
PSOURCE_DETCRIM	OtherReferral	38.11%	25.72%	27.55%	39.90%	-35.54%****
PSOURCE_DETCRIM	Prison	27.14%	35.79%	16.08%	40.43%	-11.47%
PSOURCE_DETCRIM	ProbationOrParole	23.22%	21.56%	18.12%	25.40%	-15.10%****
PSOURCE_DETCRIM	School	25.00%	29.17%	19.23%	24.06%	21.22%
PSOURCE_DETCRIM	Unknown	39.54%	21.12%	45.72%	26.82%	-21.25%****
PSOURCE_DETCRIM	UnknownCourtReferral	14.05%	16.38%	17.97%	12.63%	29.64%****
PSYPROB	No	40.54%	25.62%	31.13%	47.46%	-46.00%****
PSYPROB	Unknown	24.99%	12.34%	29.34%	20.38%	-39.43%****
PSYPROB	Yes	46.08%	37.70%	40.27%	50.44%	-25.25%****
RACE	AlaskaNative	46.97%	40.79%	38.54%	51.17%	-20.29%
RACE	AmericanIndian	43.83%	44.71%	40.79%	46.54%	-3.92%
RACE	Asian	41.91%	26.82%	36.91%	49.26%	-45.55%****
RACE	AsianOrPacificIslander	32.61%	0.00%	50.00%	28.13%	-100.00%
RACE	Black	37.52%	23.00%	28.91%	45.46%	-49.41%****
RACE	OtherSingleRace	43.48%	30.95%	42.01%	48.85%	-36.64%****
RACE	PacificIslander	36.47%	23.29%	36.24%	41.16%	-43.40%****
RACE	TwoPlusRaces	38.04%	26.97%	35.67%	43.86%	-38.52%****
RACE	Unknown	52.82%	27.73%	40.57%	34.18%	-18.87%****

Statistical significance shown at the 0.1 (*), 0.05 (**), 0.01 (***), and 0.001 (****) levels.

Group	Subgroup	Total MOUD Treatment Rate	Unhoused MOUD Treatment Rate	Dependent MOUD Treatment Rate	Independent MOUD Treatment Rate	% Difference - Unhoused from Independent Living
RACE	White	40.12%	29.60%	34.80%	44.56%	-33.56%****
REGION	Midwest	35.84%	36.56%	23.44%	41.48%	-11.86%****
REGION	Northeast	45.00%	31.95%	48.19%	48.17%	-33.68%****
REGION	South	32.79%	11.72%	20.93%	36.40%	-67.81%****
REGION	USTerritory	26.15%	15.83%	49.32%	25.88%	-38.85%**
REGION	West	47.44%	32.96%	45.54%	59.90%	-44.98%****
ROUTE1	Inhalation	39.92%	26.48%	31.43%	45.36%	-41.63%****
ROUTE1	Injection	39.57%	29.66%	35.54%	45.19%	-34.36%****
ROUTE1	Oral	39.52%	28.93%	34.10%	42.59%	-32.09%****
ROUTE1	Other	72.56%	45.09%	46.42%	60.63%	-25.63%****
ROUTE1	Smoking	36.51%	24.96%	37.30%	43.88%	-43.13%****
ROUTE1	Unknown	45.71%	23.67%	19.47%	29.77%	-20.47%**
ROUTE2	Inhalation	42.74%	32.14%	38.03%	46.60%	-31.03%****
ROUTE2	Injection	34.24%	28.34%	33.17%	38.68%	-26.72%****
ROUTE2	Oral	33.14%	24.14%	33.52%	36.24%	-33.39%****
ROUTE2	Other	35.72%	28.09%	34.04%	38.05%	-26.17%****
ROUTE2	Smoking	36.42%	28.91%	30.82%	41.88%	-30.98%****
ROUTE2	Unknown	47.52%	30.46%	37.99%	51.47%	-40.82%****
ROUTE3	Inhalation	38.08%	28.92%	34.98%	41.62%	-30.52%****
ROUTE3	Injection	33.03%	28.25%	32.64%	36.07%	-21.69%****
ROUTE3	Oral	33.95%	27.12%	31.92%	37.56%	-27.80%****
ROUTE3	Other	32.15%	25.41%	26.92%	35.65%	-28.71%***
ROUTE3	Smoking	34.42%	28.08%	28.80%	38.88%	-27.78%****
ROUTE3	Unknown	42.48%	28.88%	36.00%	47.12%	-38.70%****
SEDHPFLG	NotReported	40.47%	28.59%	34.61%	45.00%	-36.47%****
SEDHPFLG	Reported	24.24%	21.20%	20.87%	26.81%	-20.90%**
SERVICES	AmbulatoryDetox	45.77%	30.33%	63.70%	47.19%	-35.73%****
SERVICES	AmbulatoryIntensiveOutpatient	23.46%	16.41%	18.76%	27.87%	-41.12%****
SERVICES	AmbulatoryNonIntensiveOutpatient	60.75%	63.48%	51.32%	63.60%	-0.19%
SERVICES	Detox24hFreeStandingRes	14.04%	12.57%	19.41%	13.87%	-9.33%****
SERVICES	Detox24hHospitalInpatient	0.91%	1.04%	3.03%	0.80%	29.58%
SERVICES	RehabResHospitalNonDetox	25.13%	64.13%	15.38%	30.35%	111.29%****
SERVICES	RehabResLongTerm	27.89%	25.58%	28.72%	29.99%	-14.69%****
SERVICES	RehabResShortTerm	23.45%	26.37%	14.13%	25.34%	4.06%**
STFIPS	AK	49.28%	38.74%	37.21%	55.75%	-30.52%****
STFIPS	AL	29.72%	11.87%	29.66%	32.26%	-63.19%****
STFIPS	AR	33.80%	10.34%	26.16%	41.86%	-75.29%****
STFIPS	AZ	18.12%				
STFIPS	CA	58.78%	34.44%	55.53%	71.76%	-52.00%****
STFIPS	CO	36.77%	36.43%	16.65%	42.86%	-15.01%****
STFIPS	CT	30.73%	17.68%	17.63%	37.37%	-52.68%****
STFIPS	DC	6.73%	7.97%	4.83%	6.56%	21.41%
STFIPS	DE	23.86%	10.48%	15.05%	26.55%	-60.51%****
STFIPS	GA	23.14%	20.80%	12.42%	30.68%	-32.23%****
STFIPS	HI	6.88%	6.06%	3.03%	12.50%	-51.52%

Statistical significance shown at the 0.1 (*), 0.05 (**), 0.01 (***), and 0.001 (****) levels.

Group	Subgroup	Total MOUD Treatment Rate	Unhoused MOUD Treatment Rate	Dependent MOUD Treatment Rate	Independent MOUD Treatment Rate	% Difference - Unhoused from Independent Living
STFIPS	IA	35.79%	28.13%	36.63%	39.79%	-29.31%****
STFIPS	ID	2.06%	0.00%	0.00%	3.28%	-100.00%
STFIPS	IL	9.70%	6.21%	8.84%	12.84%	-51.63%****
STFIPS	IN	36.51%	24.00%	20.70%	43.15%	-44.37%****
STFIPS	KS	19.93%	12.50%	17.54%	21.88%	-42.86%
STFIPS	KY	25.41%	7.84%	22.69%	31.77%	-75.32%****
STFIPS	LA	1.85%	1.30%	2.60%	1.62%	-19.63%
STFIPS	MA	27.76%	21.63%	37.70%	29.96%	-27.81%****
STFIPS	MD	49.55%	16.11%	26.06%	57.27%	-71.86%****
STFIPS	ME	76.34%	75.32%	64.62%	78.49%	-4.04%
STFIPS	MI	56.86%	52.29%	40.74%	63.14%	-17.18%****
STFIPS	MN	53.50%	56.07%	42.41%	59.31%	-5.47%**
STFIPS	MO	14.79%	11.93%	10.28%	15.79%	-24.49%****
STFIPS	MS	2.14%	1.14%	3.23%	2.21%	-48.58%
STFIPS	MT	0.00%	0.00%	0.00%	0.00%	
STFIPS	NC	13.64%	7.67%	3.81%	14.48%	-47.02%****
STFIPS	ND	18.05%	18.75%	13.64%	18.31%	2.40%
STFIPS	NE	31.69%	12.24%	26.53%	41.51%	-70.50%****
STFIPS	NH	20.40%	21.21%	17.07%	21.38%	-0.79%
STFIPS	NJ	68.26%	68.52%	52.08%	72.17%	-5.06%****
STFIPS	NM	85.33%	90.11%	93.35%	70.63%	27.58%****
STFIPS	NV	31.94%	33.33%	13.11%	26.75%	24.60%
STFIPS	NY	46.69%	34.67%	64.20%	47.87%	-27.57%****
STFIPS	OH	32.28%	38.31%	23.44%	32.54%	17.74%****
STFIPS	OK	0.00%	0.00%	0.00%	0.00%	
STFIPS	PA	36.83%	34.00%	31.30%	43.25%	-21.40%****
STFIPS	PR	26.15%	15.83%	49.32%	25.88%	-38.85%**
STFIPS	RI	66.99%	44.56%	87.54%	63.20%	-29.49%****
STFIPS	SC	49.32%	60.00%	61.54%	46.30%	29.60%
STFIPS	SD	27.11%	0.00%	41.35%	24.66%	-100.00%***
STFIPS	TN	0.51%	0.57%	0.24%	0.52%	10.22%
STFIPS	TX	24.85%	9.39%	22.80%	39.27%	-76.09%****
STFIPS	UT	25.99%	16.06%	20.57%	33.06%	-51.44%****
STFIPS	VA	0.00%	0.00%	0.00%	0.00%	
STFIPS	VT	72.39%	66.90%	66.35%	73.86%	-9.42%***
STFIPS	WI	12.57%	5.11%	9.16%	15.41%	-66.83%****
STFIPS	WY	4.78%	11.11%	1.11%	4.55%	144.44%
STIMFLG	NotReported	40.55%	28.65%	34.90%	45.02%	-36.36%****
STIMFLG	Reported	20.62%	14.21%	15.51%	26.94%	-47.27%****
SUB1	Heroin	40.20%	28.68%	34.85%	46.25%	-38.00%****
SUB1	NoScriptMethadone	50.16%	34.21%	38.54%	52.39%	-34.70%****
SUB1	OtherOpioidSynthetics	40.91%	27.29%	32.95%	40.53%	-32.67%****
SUB2	Alcohol	28.68%	22.46%	29.64%	31.21%	-28.04%****
SUB2	Barbiturates	24.88%	15.63%	26.67%	27.05%	-42.23%
SUB2	Benzo	30.59%	22.80%	34.15%	32.66%	-30.20%****

Statistical significance shown at the 0.1 (*), 0.05 (**), 0.01 (***), and 0.001 (****) levels.

Group	Subgroup	Total MOUD Treatment Rate	Unhoused MOUD Treatment Rate	Dependent MOUD Treatment Rate	Independent MOUD Treatment Rate	% Difference - Unhoused from Independent Living
SUB2	CokeCrack	40.40%	30.49%	37.02%	45.77%	-33.39%****
SUB2	Hallucinogenics	29.60%	26.97%	20.00%	37.37%	-27.84%*
SUB2	Heroin	46.91%	39.31%	42.26%	50.26%	-21.78%****
SUB2	Inhalants	38.78%	23.08%	36.36%	48.94%	-52.84%*
SUB2	MethSpeed	29.65%	26.43%	27.75%	34.06%	-22.39%****
SUB2	NoScriptMethadone	50.39%	24.38%	41.40%	57.02%	-57.24%****
SUB2	None	42.62%	30.92%	36.92%	47.47%	-34.86%****
SUB2	OtherAmphetamines	32.40%	25.69%	30.00%	34.91%	-26.39%****
SUB2	OtherDrugs	39.51%	28.99%	40.62%	41.88%	-30.78%****
SUB2	OtherOpioidSynthetics	46.84%	36.67%	41.63%	50.55%	-27.46%****
SUB2	OtherSedatives	22.69%	18.27%	17.94%	25.84%	-29.31%**
SUB2	OtherStimulants	22.41%	17.38%	18.17%	26.74%	-35.02%****
SUB2	OtherTranqs	29.55%	26.09%	16.67%	34.12%	-23.54%
SUB2	OverTheCounter	39.30%	45.45%	34.38%	42.96%	5.81%
SUB2	PCP	31.48%	24.24%	20.27%	38.58%	-37.16%***
SUB2	PotHash	36.01%	29.61%	27.91%	39.96%	-25.91%****
SUB2	Unknown	63.19%	28.44%	46.36%	64.79%	-56.10%****
SUB3	Alcohol	32.45%	26.20%	30.77%	36.10%	-27.41%****
SUB3	Barbiturates	23.13%	10.00%	28.95%	21.92%	-54.37%
SUB3	Benzo	34.98%	28.54%	34.08%	38.16%	-25.22%****
SUB3	CokeCrack	36.34%	27.40%	35.16%	40.40%	-32.18%****
SUB3	Hallucinogenics	32.06%	29.63%	22.03%	38.87%	-23.77%*
SUB3	Heroin	32.41%	24.03%	27.57%	37.33%	-35.63%***
SUB3	Inhalants	29.51%	45.45%	42.86%	20.00%	127.27%*
SUB3	MethSpeed	29.44%	28.13%	25.99%	32.21%	-12.69%***
SUB3	NoScriptMethadone	31.86%	14.29%	24.36%	37.38%	-61.78%****
SUB3	None	41.43%	29.20%	33.97%	45.23%	-35.44%****
SUB3	OtherAmphetamines	37.28%	29.35%	33.44%	41.23%	-28.83%****
SUB3	OtherDrugs	32.67%	24.43%	26.43%	37.17%	-34.29%****
SUB3	OtherOpioidSynthetics	36.48%	30.31%	30.33%	40.75%	-25.63%****
SUB3	OtherSedatives	27.06%	26.85%	26.00%	28.54%	-5.92%
SUB3	OtherStimulants	17.93%	9.18%	12.48%	27.32%	-66.40%****
SUB3	OtherTranqs	39.13%	10.00%	33.33%	55.56%	-82.00%**
SUB3	OverTheCounter	41.21%	22.22%	15.69%	57.14%	-61.11%**
SUB3	PCP	24.94%	20.83%	23.94%	27.53%	-24.33%
SUB3	PotHash	35.45%	29.29%	29.65%	39.62%	-26.08%****
SUB3	Unknown	47.84%	28.18%	44.20%	57.72%	-51.18%****
TRNQFLG	NotReported	40.40%	28.56%	34.55%	44.92%	-36.42%****
TRNQFLG	Reported	32.02%	21.21%	20.00%	39.29%	-46.01%*
VET	NotVeteran	40.44%	28.63%	34.58%	45.78%	-37.48%****
VET	Unknown	38.48%	23.09%	31.82%	24.12%	-4.26%
VET	Veteran	44.61%	32.86%	38.62%	50.49%	-34.92%****

Statistical significance shown at the 0.1 (*), 0.05 (**), 0.01 (***), and 0.001 (****) levels.
