# Supplementary material for: Factors predicting access to medications for opioid use disorder for housed and unhoused patients: A machine learning approach
Source: PLoS One. 2024 Sep 27;19(9):e0308791. doi: 10.1371/journal.pone.0308791 (PMC11433129; doi:10.1371/journal.pone.0308791)
Supplement: S2 Appendix — (PDF) [file pone.0308791.s002.pdf]

S2 Appendix - Admission Counts and Frequencies by Subgroup

Group	Subgroup	Observations (all data)	Subgroup Frequency (all data)	Observations (balanced training data)	Subgroup Frequency (balanced training data)
ALL	ALL	524,134	100.00%	366,893	100.00%
AGE	Age12To14	177	0.03%	138	0.04%
AGE	Age15To17	617	0.12%	414	0.11%
AGE	Age18To20	6,703	1.28%	4,659	1.27%
AGE	Age21To24	35,862	6.84%	25,153	6.86%
AGE	Age25To29	106,210	20.26%	74,291	20.25%
AGE	Age30To34	111,165	21.21%	77,643	21.16%
AGE	Age35To39	82,557	15.75%	57,722	15.73%
AGE	Age40To44	51,264	9.78%	35,915	9.79%
AGE	Age45To49	40,611	7.75%	28,637	7.81%
AGE	Age50To54	37,784	7.21%	26,416	7.20%
AGE	Age55To64	43,497	8.30%	30,493	8.31%
AGE	Age65Plus	7,687	1.47%	5,412	1.48%
ALCDRUG	AlcoholAndDrugs	66,488	12.69%	46,568	12.69%
ALCDRUG	OtherDrugs	457,646	87.31%	320,325	87.31%
ALCFLG	NotReported	457,646	87.31%	320,325	87.31%
ALCFLG	Reported	66,488	12.69%	46,568	12.69%
AMPHFLG	NotReported	519,658	99.15%	363,789	99.15%
AMPHFLG	Reported	4,476	0.85%	3,104	0.85%
ARRESTS	0Arrest	447,407	85.36%	313,254	85.38%
ARRESTS	1Arrest	30,255	5.77%	21,121	5.76%
ARRESTS	2PlusArrest	5,333	1.02%	3,736	1.02%
ARRESTS	Unknown	41,139	7.85%	28,782	7.84%
BARBFLG	NotReported	523,799	99.94%	366,656	99.94%
BARBFLG	Reported	335	0.06%	237	0.06%
BENZFLG	NotReported	482,491	92.05%	337,675	92.04%
BENZFLG	Reported	41,643	7.95%	29,218	7.96%
CBSA2010	*Excluded due to large number of subgroups (272)*				
COKEFLG	NotReported	391,782	74.75%	274,439	74.80%
COKEFLG	Reported	132,352	25.25%	92,454	25.20%
DAYWAIT	0DaysWait	219,010	41.79%	153,487	41.83%
DAYWAIT	15To30DaysWait	6,470	1.23%	4,455	1.21%
DAYWAIT	1To7DaysWait	67,515	12.88%	47,311	12.90%
DAYWAIT	31PlusDaysWait	4,214	0.80%	2,923	0.80%
DAYWAIT	8To14DaysWait	9,515	1.82%	6,661	1.82%
DAYWAIT	Unknown	217,410	41.48%	152,056	41.44%
DIVISION	EastNorthCentral	67,030	12.79%	46,664	12.72%
DIVISION	EastSouthCentral	20,753	3.96%	14,596	3.98%
DIVISION	MiddleAtlantic	131,658	25.12%	92,261	25.15%
DIVISION	Mountain	32,372	6.18%	22,685	6.18%
DIVISION	NewEngland	81,590	15.57%	57,013	15.54%
DIVISION	Pacific	43,881	8.37%	30,765	8.39%
DIVISION	SouthAtlantic	108,594	20.72%	76,074	20.73%
DIVISION	USTerritories	1,170	0.22%	825	0.22%

Group	Subgroup	Admission Count	Subgroup Frequency	Total MOUD Treatment Rate	MOUD Access Rate - Total
DIVISION	WestNorthCentral	22,513	4.30%	15,814	4.31%
DIVISION	WestSouthCentral	14,573	2.78%	10,196	2.78%
DSMCRIT	ADDOrBehaviorDisorder	38	0.01%	29	0.01%
DSMCRIT	AlcoholAbuse	920	0.18%	670	0.18%
DSMCRIT	AlcoholDependence	6,216	1.19%	4,379	1.19%
DSMCRIT	AlcoholDisorder	103	0.02%	70	0.02%
DSMCRIT	AlcoholIntoxication	134	0.03%	98	0.03%
DSMCRIT	AnxietyDisorder	346	0.07%	247	0.07%
DSMCRIT	BipolarDisorder	329	0.06%	227	0.06%
DSMCRIT	CannabisAbuse	817	0.16%	552	0.15%
DSMCRIT	CannabisDependence	1,956	0.37%	1,366	0.37%
DSMCRIT	CocaineAbuse	374	0.07%	255	0.07%
DSMCRIT	CocaineDependence	2,838	0.54%	1,984	0.54%
DSMCRIT	DepressiveDisorder	697	0.13%	503	0.14%
DSMCRIT	OpioidAbuse	13,275	2.53%	9,252	2.52%
DSMCRIT	OpioidDependence	309,818	59.11%	216,891	59.12%
DSMCRIT	OtherMentalHealthCondition	44,710	8.53%	31,309	8.53%
DSMCRIT	OtherSubstanceAbuse	1,030	0.20%	732	0.20%
DSMCRIT	OtherSubstanceDependence	5,553	1.06%	3,900	1.06%
DSMCRIT	SchizopheniaOrPsychDisorder	101	0.02%	73	0.02%
DSMCRIT	SubstanceDisorder	7,914	1.51%	5,548	1.51%
DSMCRIT	Unknown	126,965	24.22%	88,808	24.21%
EDUC	1To3yCollege	88,172	16.82%	61,670	16.81%
EDUC	4yCollegePlus	26,573	5.07%	18,585	5.07%
EDUC	Grade12OrGED	253,442	48.35%	177,621	48.41%
EDUC	Grade8OrLess	29,059	5.54%	20,232	5.51%
EDUC	Grade9To11	96,982	18.50%	67,849	18.49%
EDUC	Unknown	29,906	5.71%	20,936	5.71%
EMPLOY_DETNLF	FullTime	69,245	13.21%	48,490	13.22%
EMPLOY_DETNLF	Homemaker	3,326	0.63%	2,349	0.64%
EMPLOY_DETNLF	InstitutionResident	9,426	1.80%	6,592	1.80%
EMPLOY_DETNLF	OtherNotInLaborForce	134,529	25.67%	94,040	25.63%
EMPLOY_DETNLF	PartTime	30,060	5.74%	21,081	5.75%
EMPLOY_DETNLF	RetiredOrDisabled	38,813	7.41%	27,130	7.39%
EMPLOY_DETNLF	Student	1,784	0.34%	1,193	0.33%
EMPLOY_DETNLF	Unemployed	191,417	36.52%	134,031	36.53%
EMPLOY_DETNLF	Unknown	23,056	4.40%	16,200	4.42%
EMPLOY_DETNLF	UnknownNotInLaborForce	22,478	4.29%	15,787	4.30%
ETHNIC	CubanOrOther	13,234	2.52%	9,278	2.53%
ETHNIC	Mexican	14,597	2.78%	10,238	2.79%
ETHNIC	NonHispanic	437,559	83.48%	306,223	83.46%
ETHNIC	PuertoRican	30,009	5.73%	21,045	5.74%
ETHNIC	Unknown	15,955	3.04%	11,116	3.03%
ETHNIC	UnspecifiedHispanic	12,780	2.44%	8,993	2.45%
FREQ_ATND_SELF_HELP	1To3TimesPastMonth	33,748	6.44%	23,713	6.46%

Group	Subgroup	Admission Count	Subgroup Frequency	Total MOUD Treatment Rate	MOUD Access Rate - Total
FREQ_ATND_SELF_HELP	4To7TimesPastMonth	20,638	3.94%	14,596	3.98%
FREQ_ATND_SELF_HELP	8To30TimesPastMonth	41,755	7.97%	29,184	7.95%
FREQ_ATND_SELF_HELP	NoAttendance	332,025	63.35%	232,081	63.26%
FREQ_ATND_SELF_HELP	SomeAttendance	32,623	6.22%	22,946	6.25%
FREQ_ATND_SELF_HELP	Unknown	63,345	12.09%	44,373	12.09%
FREQ1	DailyUse	305,825	58.35%	214,063	58.34%
FREQ1	NoUsePastMonth	121,230	23.13%	84,850	23.13%
FREQ1	SomeUse	87,469	16.69%	61,243	16.69%
FREQ1	Unknown	9,610	1.83%	6,737	1.84%
FREQ2	DailyUse	136,621	26.07%	95,499	26.03%
FREQ2	NoUsePastMonth	83,407	15.91%	58,511	15.95%
FREQ2	SomeUse	104,598	19.96%	73,133	19.93%
FREQ2	Unknown	199,508	38.06%	139,750	38.09%
FREQ3	DailyUse	49,090	9.37%	34,240	9.33%
FREQ3	NoUsePastMonth	42,225	8.06%	29,630	8.08%
FREQ3	SomeUse	47,064	8.98%	33,117	9.03%
FREQ3	Unknown	385,755	73.60%	269,906	73.57%
FRSTUSE1	11yLess	6,208	1.18%	4,331	1.18%
FRSTUSE1	12To14y	34,248	6.53%	23,944	6.53%
FRSTUSE1	15To17y	79,970	15.26%	55,751	15.20%
FRSTUSE1	18To20y	106,956	20.41%	74,898	20.41%
FRSTUSE1	21To24y	91,979	17.55%	64,341	17.54%
FRSTUSE1	25To29y	81,576	15.56%	57,161	15.58%
FRSTUSE1	30yPlus	104,564	19.95%	73,376	20.00%
FRSTUSE1	Unknown	18,633	3.56%	13,091	3.57%
FRSTUSE2	11yLess	12,666	2.42%	8,888	2.42%
FRSTUSE2	12To14y	50,914	9.71%	35,591	9.70%
FRSTUSE2	15To17y	73,547	14.03%	51,518	14.04%
FRSTUSE2	18To20y	58,474	11.16%	40,773	11.11%
FRSTUSE2	21To24y	39,113	7.46%	27,387	7.46%
FRSTUSE2	25To29y	35,906	6.85%	25,172	6.86%
FRSTUSE2	30yPlus	42,233	8.06%	29,640	8.08%
FRSTUSE2	Unknown	211,281	40.31%	147,924	40.32%
FRSTUSE3	11yLess	9,602	1.83%	6,721	1.83%
FRSTUSE3	12To14y	33,425	6.38%	23,293	6.35%
FRSTUSE3	15To17y	35,647	6.80%	25,007	6.82%
FRSTUSE3	18To20y	20,570	3.92%	14,463	3.94%
FRSTUSE3	21To24y	11,466	2.19%	8,101	2.21%
FRSTUSE3	25To29y	10,127	1.93%	7,137	1.95%
FRSTUSE3	30yPlus	12,803	2.44%	8,962	2.44%
FRSTUSE3	Unknown	390,494	74.50%	273,209	74.47%
GENDER	Female	192,792	36.78%	134,938	36.78%
GENDER	Male	330,818	63.12%	231,599	63.12%
GENDER	Unknown	524	0.10%	356	0.10%
HALLFLG	NotReported	523,183	99.82%	366,228	99.82%

Group	Subgroup	Admission Count	Subgroup Frequency	Total MOUD Treatment Rate	MOUD Access Rate - Total
HALLFLG	Reported	951	0.18%	665	0.18%
HERFLG	NotReported	104,841	20.00%	73,675	20.08%
HERFLG	Reported	419,293	80.00%	293,218	79.92%
HLTHINS	Medicaid	187,253	35.73%	131,041	35.72%
HLTHINS	MedicareOrOther	18,456	3.52%	12,948	3.53%
HLTHINS	None	62,769	11.98%	44,098	12.02%
HLTHINS	PrivateInsurance	12,202	2.33%	8,578	2.34%
HLTHINS	Unknown	243,454	46.45%	170,228	46.40%
IDU	IDU	260,856	49.77%	182,435	49.72%
IDU	NoIDU	263,278	50.23%	184,458	50.28%
INHFLG	NotReported	523,975	99.97%	366,775	99.97%
INHFLG	Reported	159	0.03%	118	0.03%
LIVARAG	DependLiving	83,668	15.96%	58,668	15.99%
LIVARAG	Unhoused	85,824	16.37%	228,697	62.33%
LIVARAG	IndependentLiving	326,715	62.33%	60,051	16.37%
LIVARAG	Unknown	27,927	5.33%	19,477	5.31%
MARFLG	NotReported	437,447	83.46%	306,188	83.45%
MARFLG	Reported	86,687	16.54%	60,705	16.55%
MARSTAT	DivorcedOrWidowed	50,256	9.59%	35,190	9.59%
MARSTAT	NeverMarried	296,739	56.62%	207,610	56.59%
MARSTAT	NowMarried	49,090	9.37%	34,416	9.38%
MARSTAT	Separated	21,710	4.14%	15,275	4.16%
MARSTAT	Unknown	106,339	20.29%	74,402	20.28%
METHFLG	NotReported	519,399	99.10%	363,609	99.10%
METHFLG	Reported	4,735	0.90%	3,284	0.90%
METHUSE	0	312,391	59.60%	148,230	40.40%
METHUSE	1	211,743	40.40%	218,663	59.60%
MTHAMFLG	NotReported	455,130	86.83%	318,468	86.80%
MTHAMFLG	Reported	69,004	13.17%	48,425	13.20%
NOPRIOR	0PriorTreatments	147,506	28.14%	103,177	28.12%
NOPRIOR	1PriorTreatments	83,008	15.84%	58,337	15.90%
NOPRIOR	2PriorTreatments	64,121	12.23%	44,751	12.20%
NOPRIOR	3PriorTreatments	44,771	8.54%	31,331	8.54%
NOPRIOR	4PriorTreatments	28,300	5.40%	19,846	5.41%
NOPRIOR	5PlusPriorTreatments	106,841	20.38%	74,733	20.37%
NOPRIOR	Unknown	49,587	9.46%	34,718	9.46%
OPSYNFLG	NotReported	384,914	73.44%	269,130	73.35%
OPSYNFLG	Reported	139,220	26.56%	97,763	26.65%
OTCFLG	NotReported	523,740	99.92%	366,618	99.93%
OTCFLG	Reported	394	0.08%	275	0.07%
OTHERFLG	NotReported	515,107	98.28%	360,572	98.28%
OTHERFLG	Reported	9,027	1.72%	6,321	1.72%
PCPFLG	NotReported	523,203	99.82%	366,269	99.83%
PCPFLG	Reported	931	0.18%	624	0.17%
PREG	NotPregnant	173,323	33.07%	121,342	33.07%

Group	Subgroup	Admission Count	Subgroup Frequency	Total MOUD Treatment Rate	MOUD Access Rate - Total
PREG	Pregnant	7,473	1.43%	5,235	1.43%
PREG	Unknown	343,338	65.51%	240,316	65.50%
PRIMINC	None	132,557	25.29%	92,860	25.31%
PRIMINC	Other	64,386	12.28%	44,961	12.25%
PRIMINC	PublicAssist	37,355	7.13%	25,960	7.08%
PRIMINC	RetireOrDisable	27,375	5.22%	19,337	5.27%
PRIMINC	Unknown	186,176	35.52%	130,174	35.48%
PRIMINC	Wages	76,285	14.55%	53,601	14.61%
PRIMPAY	Medicaid	150,150	28.65%	105,092	28.64%
PRIMPAY	Medicare	2,867	0.55%	2,005	0.55%
PRIMPAY	NoCharge	5,816	1.11%	4,118	1.12%
PRIMPAY	Other	7,365	1.41%	5,153	1.40%
PRIMPAY	OtherGovtPayment	44,295	8.45%	31,075	8.47%
PRIMPAY	PrivateInsurance	7,101	1.35%	4,973	1.36%
PRIMPAY	SelfPay	8,206	1.57%	5,774	1.57%
PRIMPAY	Unknown	298,334	56.92%	208,703	56.88%
PSOURCE_DETCRIM	Adjudication	3,457	0.66%	2,435	0.66%
PSOURCE_DETCRIM	Court	11,527	2.20%	8,120	2.21%
PSOURCE_DETCRIM	DUI	1,129	0.22%	3,221	0.88%
PSOURCE_DETCRIM	DiversionaryProgram	4,590	0.88%	44,612	12.16%
PSOURCE_DETCRIM	DrugCareProvider	63,868	12.19%	792	0.22%
PSOURCE_DETCRIM	Employer	1,601	0.31%	1,099	0.30%
PSOURCE_DETCRIM	Individual	299,582	57.16%	209,743	57.17%
PSOURCE_DETCRIM	Other	6,743	1.29%	4,719	1.29%
PSOURCE_DETCRIM	OtherHealthCareProvider	27,048	5.16%	18,790	5.12%
PSOURCE_DETCRIM	OtherLegalEntity	3,012	0.57%	2,094	0.57%
PSOURCE_DETCRIM	OtherReferral	45,594	8.70%	32,011	8.72%
PSOURCE_DETCRIM	Prison	2,929	0.56%	2,052	0.56%
PSOURCE_DETCRIM	ProbationOrParole	24,201	4.62%	16,958	4.62%
PSOURCE_DETCRIM	School	188	0.04%	133	0.04%
PSOURCE_DETCRIM	Unknown	10,708	2.04%	7,558	2.06%
PSOURCE_DETCRIM	UnknownCourtReferral	17,957	3.43%	12,556	3.42%
PSYPROB	No	246,594	47.05%	172,843	47.11%
PSYPROB	Unknown	76,384	14.57%	53,463	14.57%
PSYPROB	Yes	201,156	38.38%	140,587	38.32%
RACE	AlaskaNative	479	0.09%	344	0.09%
RACE	AmericanIndian	5,909	1.13%	4,140	1.13%
RACE	Asian	2,350	0.45%	1,634	0.45%
RACE	AsianOrPacificIslander	46	0.01%	33	0.01%
RACE	Black	82,583	15.76%	57,851	15.77%
RACE	OtherSingleRace	42,817	8.17%	29,874	8.14%
RACE	PacificIslander	1,349	0.26%	968	0.26%
RACE	TwoPlusRaces	8,442	1.61%	5,913	1.61%
RACE	Unknown	16,470	3.14%	11,532	3.14%
RACE	White	363,689	69.39%	254,604	69.39%

Group	Subgroup	Admission Count	Subgroup Frequency	Total MOUD Treatment Rate	MOUD Access Rate - Total
REGION	Midwest	89,543	17.08%	62,478	17.03%
REGION	Northeast	213,248	40.69%	149,274	40.69%
REGION	South	143,920	27.46%	100,866	27.49%
REGION	USTerritory	1,170	0.22%	825	0.22%
REGION	West	76,253	14.55%	53,450	14.57%
ROUTE1	Inhalation	153,783	29.34%	107,545	29.31%
ROUTE1	Injection	253,372	48.34%	177,184	48.29%
ROUTE1	Oral	69,816	13.32%	49,085	13.38%
ROUTE1	Other	13,427	2.56%	9,451	2.58%
ROUTE1	Smoking	28,877	5.51%	20,265	5.52%
ROUTE1	Unknown	4,859	0.93%	3,363	0.92%
ROUTE2	Inhalation	43,816	8.36%	30,581	8.34%
ROUTE2	Injection	62,320	11.89%	43,501	11.86%
ROUTE2	Oral	85,476	16.31%	59,890	16.32%
ROUTE2	Other	2,724	0.52%	1,933	0.53%
ROUTE2	Smoking	129,279	24.67%	90,539	24.68%
ROUTE2	Unknown	200,519	38.26%	140,449	38.28%
ROUTE3	Inhalation	14,089	2.69%	9,919	2.70%
ROUTE3	Injection	12,670	2.42%	8,849	2.41%
ROUTE3	Oral	47,655	9.09%	33,395	9.10%
ROUTE3	Other	1,067	0.20%	736	0.20%
ROUTE3	Smoking	60,903	11.62%	42,728	11.65%
ROUTE3	Unknown	387,750	73.98%	271,266	73.94%
SEDHPFLG	NotReported	521,919	99.58%	365,349	99.58%
SEDHPFLG	Reported	2,215	0.42%	1,544	0.42%
SERVICES	AmbulatoryDetox	4,973	0.95%	3,437	0.94%
SERVICES	AmbulatoryIntensiveOutpatient	57,703	11.01%	40,458	11.03%
SERVICES	AmbulatoryNonIntensiveOutpatient	262,944	50.17%	184,053	50.17%
SERVICES	Detox24hFreeStandingRes	97,123	18.53%	67,882	18.50%
SERVICES	Detox24hHospitalInpatient	12,470	2.38%	8,779	2.39%
SERVICES	RehabResHospitalNonDetox	756	0.14%	538	0.15%
SERVICES	RehabResLongTerm	35,301	6.74%	24,709	6.73%
SERVICES	RehabResShortTerm	52,864	10.09%	37,037	10.09%
STFIPS	AK	1,603	0.31%	1,133	0.31%
STFIPS	AL	5,774	1.10%	4,106	1.12%
STFIPS	AR	1,947	0.37%	1,321	0.36%
STFIPS	AZ	10,189	1.94%	7,054	1.92%
STFIPS	CA	42,031	8.02%	29,470	8.03%
STFIPS	CO	13,102	2.50%	9,212	2.51%
STFIPS	CT	23,274	4.44%	16,341	4.45%
STFIPS	DC	1,560	0.30%	1,094	0.30%
STFIPS	DE	8,910	1.70%	6,297	1.72%
STFIPS	GA	2,965	0.57%	2,084	0.57%
STFIPS	HI	247	0.05%	162	0.04%
STFIPS	IA	2,174	0.41%	1,508	0.41%

Group	Subgroup	Admission Count	Subgroup Frequency	Total MOUD Treatment Rate	MOUD Access Rate - Total
STFIPS	ID	97	0.02%	70	0.02%
STFIPS	IL	16,255	3.10%	11,384	3.10%
STFIPS	IN	7,162	1.37%	4,988	1.36%
STFIPS	KS	296	0.06%	219	0.06%
STFIPS	KY	6,463	1.23%	4,566	1.24%
STFIPS	LA	2,111	0.40%	1,485	0.40%
STFIPS	MA	44,251	8.44%	30,933	8.43%
STFIPS	MD	72,675	13.87%	50,822	13.85%
STFIPS	ME	3,728	0.71%	2,584	0.70%
STFIPS	MI	29,140	5.56%	20,281	5.53%
STFIPS	MN	10,138	1.93%	7,085	1.93%
STFIPS	MO	8,889	1.70%	6,308	1.72%
STFIPS	MS	1,260	0.24%	884	0.24%
STFIPS	MT	106	0.02%	76	0.02%
STFIPS	NC	15,707	3.00%	11,010	3.00%
STFIPS	ND	133	0.03%	90	0.02%
STFIPS	NE	385	0.07%	264	0.07%
STFIPS	NH	1,157	0.22%	815	0.22%
STFIPS	NJ	30,941	5.90%	21,749	5.93%
STFIPS	NM	3,006	0.57%	2,130	0.58%
STFIPS	NV	648	0.12%	464	0.13%
STFIPS	NY	90,297	17.23%	63,161	17.22%
STFIPS	OH	8,411	1.60%	5,810	1.58%
STFIPS	OK	2,574	0.49%	1,804	0.49%
STFIPS	PA	10,420	1.99%	7,351	2.00%
STFIPS	PR	1,170	0.22%	825	0.22%
STFIPS	RI	5,768	1.10%	4,001	1.09%
STFIPS	SC	73	0.01%	46	0.01%
STFIPS	SD	498	0.10%	340	0.09%
STFIPS	TN	7,256	1.38%	5,040	1.37%
STFIPS	TX	7,941	1.52%	5,586	1.52%
STFIPS	UT	4,575	0.87%	3,214	0.88%
STFIPS	VA	6,704	1.28%	4,721	1.29%
STFIPS	VT	3,412	0.65%	2,339	0.64%
STFIPS	WI	6,062	1.16%	4,201	1.15%
STFIPS	WY	649	0.12%	465	0.13%
STIMFLG	NotReported	520,158	99.24%	364,061	99.23%
STIMFLG	Reported	3,976	0.76%	2,832	0.77%
SUB1	Heroin	409,962	78.22%	286,711	78.15%
SUB1	NoScriptMethadone	2,444	0.47%	1,707	0.47%
SUB1	OtherOpioidSynthetics	111,728	21.32%	78,475	21.39%
SUB2	Alcohol	40,780	7.78%	28,529	7.78%
SUB2	Barbiturates	201	0.04%	135	0.04%
SUB2	Benzo	27,034	5.16%	18,998	5.18%
SUB2	CokeCrack	108,785	20.76%	75,920	20.69%

Group	Subgroup	Admission Count	Subgroup Frequency	Total MOUD Treatment Rate	MOUD Access Rate - Total
SUB2	Hallucinogenics	402	0.08%	284	0.08%
SUB2	Heroin	8,103	1.55%	5,681	1.55%
SUB2	Inhalants	98	0.02%	70	0.02%
SUB2	MethSpeed	59,329	11.32%	41,606	11.34%
SUB2	NoScriptMethadone	1,522	0.29%	104,523	28.49%
SUB2	None	149,146	28.46%	1,059	0.29%
SUB2	OtherAmphetamines	2,821	0.54%	1,963	0.54%
SUB2	OtherDrugs	3,812	0.73%	2,660	0.73%
SUB2	OtherOpioidSynthetics	21,577	4.12%	15,126	4.12%
SUB2	OtherSedatives	1,428	0.27%	993	0.27%
SUB2	OtherStimulants	2,392	0.46%	1,711	0.47%
SUB2	OtherTranqs	132	0.03%	89	0.02%
SUB2	OverTheCounter	229	0.04%	151	0.04%
SUB2	PCP	486	0.09%	329	0.09%
SUB2	PotHash	45,382	8.66%	31,727	8.65%
SUB2	Unknown	50,475	9.63%	35,339	9.63%
SUB3	Alcohol	25,708	4.90%	18,039	4.92%
SUB3	Barbiturates	134	0.03%	102	0.03%
SUB3	Benzo	14,609	2.79%	10,220	2.79%
SUB3	CokeCrack	23,567	4.50%	16,534	4.51%
SUB3	Hallucinogenics	549	0.10%	381	0.10%
SUB3	Heroin	1,228	0.23%	826	0.23%
SUB3	Inhalants	61	0.01%	48	0.01%
SUB3	MethSpeed	9,675	1.85%	6,819	1.86%
SUB3	NoScriptMethadone	769	0.15%	226,024	61.60%
SUB3	None	323,239	61.67%	518	0.14%
SUB3	OtherAmphetamines	1,655	0.32%	1,141	0.31%
SUB3	OtherDrugs	5,215	0.99%	3,661	1.00%
SUB3	OtherOpioidSynthetics	5,915	1.13%	4,162	1.13%
SUB3	OtherSedatives	787	0.15%	551	0.15%
SUB3	OtherStimulants	1,584	0.30%	1,121	0.31%
SUB3	OtherTranqs	46	0.01%	37	0.01%
SUB3	OverTheCounter	165	0.03%	124	0.03%
SUB3	PCP	445	0.08%	295	0.08%
SUB3	PotHash	41,305	7.88%	28,978	7.90%
SUB3	Unknown	67,478	12.87%	47,312	12.90%
TRNQFLG	NotReported	523,956	99.97%	366,767	99.97%
TRNQFLG	Reported	178	0.03%	126	0.03%
VET	NotVeteran	484,444	92.43%	339,218	92.46%
VET	Unknown	30,596	5.84%	21,326	5.81%
VET	Veteran	9,094	1.74%	6,349	1.73%
