# Supplementary material for: Factors predicting access to medications for opioid use disorder for housed and unhoused patients: A machine learning approach
Source: PLoS One. 2024 Sep 27;19(9):e0308791. doi: 10.1371/journal.pone.0308791 (PMC11433129; doi:10.1371/journal.pone.0308791)
Supplement: S3 Appendix — (PDF) [file pone.0308791.s003.pdf]

S3 Appendix - Full Feature List

	Feature name	Feature short definition	Feature category	Feature importance	Share of feature importance
1	AGE	Age at admission	Demographic	0.0975	1.94%
2	ALCDRUG	Substance use type	Substance use history	0.0044	0.09%
3	ALCFLG	Alcohol reported at admission	Substance use history	0.0284	0.56%
4	AMPHFLG	Other amphetamines reported at admission	Substance use history	0.0020	0.04%
5	ARRESTS	Arrests in the past 30 days	Personal history	0.0882	1.76%
6	BARBFLG	Barbiturates reported at admission	Substance use history	0.0000	0.00%
7	BENZFLG	Benzodiazepines reported at admission	Substance use history	0.0075	0.15%
8	CBSA2010	Core based statistical area (metro and micro areas)	Geographic	0.1754	3.49%
9	COKEFLG	Cocaine/crack reported at admission	Substance use history	0.0090	0.18%
10	DAYWAIT	Days waiting to enter substance use treatment	Coordination of care	0.0845	1.68%
11	DIVISION	Census division	Geographic	0.2901	5.77%
12	DSMCRIT	DSM diagnosis (SuDS 4 SuDS 19)	Medical history	0.1084	2.16%
13	EDUC	Education	Personal history	0.0405	0.81%
14	EMPLOY_DETNLF	Employment status (combined with not in labor force)	Economic	0.0768	1.53%
15	ETHNIC	Ethnicity	Demographic	0.0228	0.45%
16	FREQ_ATND_SELF_HELP	Attendance at substance use self-help groups in the past 30 days	Medical history	0.0952	1.89%
17	FREQ1	Frequency of use (primary)	Substance use history	0.1729	3.44%
18	FREQ2	Frequency of use (secondary)	Substance use history	0.0466	0.93%
19	FREQ3	Frequency of use (primary)	Substance use history	0.0164	0.33%
20	FRSTUSE1	Age at first use (primary)	Substance use history	0.0599	1.19%
21	FRSTUSE2	Age at first use (secondary)	Substance use history	0.0238	0.47%
22	FRSTUSE3	Age at first use (tertiary)	Substance use history	0.0149	0.30%
23	GENDER	Gender	Demographic	0.0324	0.64%
24	HALLFLG	Hallucinogens reported at admission	Substance use history	0.0002	0.00%
25	HERFLG	Heroin reported at admission	Substance use history	0.0442	0.88%
26	HLTHINS	Health insurance	Economic	0.1002	1.99%
27	IDU	Current IV drug use reported at admission	Substance use history	0.0354	0.71%
28	INHFLG	Inhalants reported at admission	Substance use history	0.0000	0.00%
29	LIVARAG	Living arrangement	Economic	0.0761	1.51%
30	MARFLG	Marijuana/hashish reported at admission	Substance use history	0.0286	0.57%
31	MARSTAT	Marital status	Personal history	0.0504	1.00%
32	METHFLG	Non-rx methadone reported at admission	Substance use history	0.0030	0.06%
33	MTHAMFLG	Methamphetamine reported at admission	Substance use history	0.0477	0.95%
34	NOPRIOR	Previous substance use treatment episodes	Medical history	0.2217	4.41%
35	OPSYNFLG	Other opiates/synthetics reported at admission	Substance use history	0.0138	0.27%
36	OTCFLG	Over-the-counter medication reported at admission	Substance use history	0.0001	0.00%
37	OTHERFLG	Other drug reported at admission	Substance use history	0.0040	0.08%
38	PCPFLG	PCP reported at admission	Substance use history	0.0001	0.00%
39	PREG	Pregnant at admission	Medical history	0.0313	0.62%
40	PRIMINC	Source of income/support	Economic	0.0817	1.63%
41	PRIMPAY	Payment source, primary (expected or actual)	Economic	0.0878	1.75%
42	PSOURCE_DETCRIM	Referral source (combined with detailed criminal justice referral)	Coordination of care	0.3386	6.74%
43	PSYPROB	Co-occurring mental and substance use disorders	Medical history	0.0911	1.81%
44	RACE	Race	Demographic	0.0385	0.77%
45	REGION	Census region	Geographic	0.3526	7.02%
46	ROUTE1	Route of administration (primary)	Substance use history	0.0323	0.64%
47	ROUTE2	Route of administration (secondary)	Substance use history	0.0346	0.69%
48	ROUTE3	Route of administration (tertiary)	Substance use history	0.0115	0.23%
49	SEDHPFLG	Other sedatives/hypnotics reported at admission	Substance use history	0.0010	0.02%
50	SERVICES	Type of treatment service/setting	Coordination of care	1.1726	23.34%
51	STFIPS	Census state FIPS code	Geographic	0.4748	9.45%
52	STIMFLG	Other stimulants reported at admission	Substance use history	0.0017	0.03%
53	SUB1	Substance use (primary)	Substance use history	0.0394	0.78%
54	SUB2	Substance use (secondary)	Substance use history	0.0783	1.56%
55	SUB3	Substance use (tertiary)	Substance use history	0.0265	0.53%
56	TRNQFLG	Other tranquilizers reported at admission	Substance use history	0.0000	0.00%
57	VET	Veteran status	Personal history	0.0074	0.15%
